# Design, synthesis, and biological investigation of oxadiazolyl, thiadiazolyl, and pyrimidinyl linked antipyrine derivatives as potential non-acidic anti-inflammatory agents

**DOI:** 10.1080/14756366.2022.2162511

**Published:** 2023-01-12

**Authors:** Mohammad M. Al-Sanea, Abdelrahman Hamdi, Simone Brogi, Samar S. Tawfik, Dina I. A. Othman, Mahmoud Elshal, Hidayat Ur Rahman, Della G. T. Parambi, Rehab M. Elbargisy, Samy Selim, Ehab M. Mostafa, Ahmed A. B. Mohamed

**Affiliations:** a Department of Pharmaceutical Chemistry, College of Pharmacy, Jouf University, Sakaka, Saudi Arabia; b Department of Pharmaceutical Organic Chemistry, Faculty of Pharmacy, Mansoura University, Mansoura, Egypt; c Department of Pharmacy, University of Pisa, Pisa, Italy; d Department of Pharmacology and Toxicology, Faculty of Pharmacy, Mansoura University, Mansoura, Egypt; e Department of Clinical Pharmacy, College of Pharmacy, Jouf University, Sakaka, Saudi Arabia; f Department of Pharmaceutics, College of Pharmacy, Jouf University, Sakaka, Saudi Arabia; g Department of Clinical Laboratory Sciences, College of Applied Medical Sciences, Jouf University, Sakaka, Saudi Arabia; h Department of Pharmacognosy, College of Pharmacy, Jouf University, Sakaka, Saudi Arabia; i Department of Medicinal Chemistry, Faculty of Pharmacy, Mansoura University, Mansoura, Egypt

**Keywords:** Antipyrine, oxadiazole, thiadiazole, pyrimidine, anti-inflammatory

## Abstract

A novel series of 12 antipyrine derivatives containing 1,3,4-oxadiazoles (**4a-d)**, 1,3,4-thiadiazoles (**6a**-**d)**, and pyrimidines (**8a**-**d)**, was preparedand assessed for its potential *in vitro* COX-2 inhibitors. Compared to Celecoxib, compounds **4b-d** and **8d** were the most potent derivatives c with a half-maximal inhibitory concentration range of 53–69 nM. Considering COX-2 selectivity index, compounds **4 b** and **4c** were chosen among these most potent derivatives for further investigation. The *in vivo* ability of compounds **4 b** and **4c** to counteract carrageenan-induced paw edoema has been assessed and their potential underlying mechanisms have been elucidated and the results have been further validated using molecular docking simulations.

## Introduction

Because of their anti-inflammatory, anti-pyretic, and analgesic properties, nonsteroidal anti-inflammatory drugs (NSAIDs) are among the most often used treatments worldwide.^1^ They are considered as the drugs of choice in numerous inflammatory disorders like rheumatoid arthritis (RA), osteoarthritis, and tendonitis. Other medical indications include muscle aches, backaches, and dental pain. Moreover, they are routinely prescribed as a palliative therapy in cases of cancer, gout, and menstrual cramps. They induce their pharmacological effects *via* the inhibition of cyclooxygenase (COX) enzymes, which in turn suppress the formation of the pro-inflammatory mediators, prostaglandins (PGs), from arachidonic acid.^2^^,^^3^ Constitutive COX-1 and inducible COX-2 are the two mainly described COX isozymes in literature.^4^ It is worth saying that COX-2 is linked to several pathological diseases, including cancer and inflammation. Both isoforms share a 65% sequence homology and their active sites are approximately indistinguishable. The substitution of valine 523 in COX-2 active site for the relatively bulkier isoleucine residue in COX-1, produces an extra side pocket with limited access to this additional binding pocket. It is worth to mention that this difference can be exploited by COX-2 selective ligands such as celecoxib.

Long-term use of non-selective NSAIDs can cause gastrointestinal discomfort, bleeding, ulceration, and other undesirable side effects.^5–7^ The primary reason for these negative pharmacological effects is the inhibitory effect on the gastroprotective prostanoids produced by COX-1 enzymes in the gastrointestinal tract. As a result, there is a growing interest in the development of powerful and selective COX-2 inhibitors with improved gastro-intestinal safety profiles.^8^ Furthermore, the use of non-acidic NSAIDs may reduce their direct ulcerogenic properties.^9^^,^^10^

Recently, antipyrine structural motif is a valuable tool that has attracted considerable attention for designing new medications for the management of various kinds of diseases. Over the past decades, antipyrine-containing compounds have caught researchers’ interest looking for new potential NSAIDs since they act as analgesics that help to decrease the pain and inflammation in cases of arthritis, gout, and others.^11–16^

**Figure 1.**
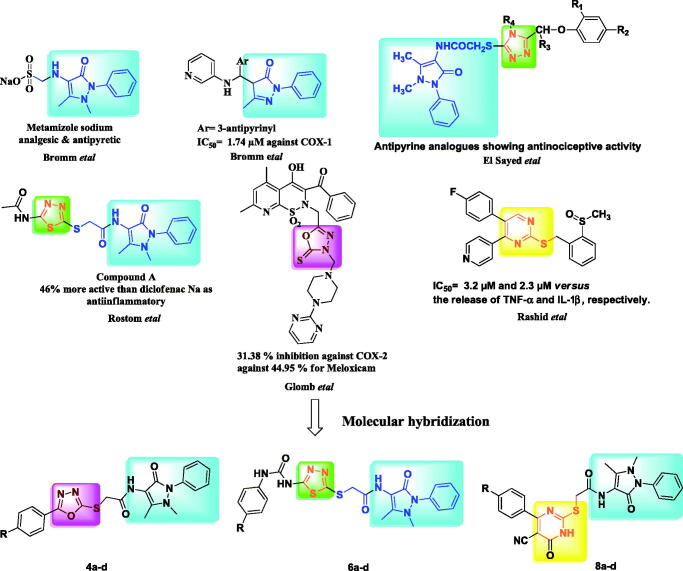
Chemical structures of certain reported antipyrine, oxadiazolyl, thiadiazolyl, pyrimidinyl analogues and our designed compounds (**4**,**6**,**8**).

Reviewing the literature, we observed the incorporation of 1,3,4-oxadiazole,^17–20^ 1,3,4-thiadiazole,^17^^,^^21–23^ and pyrimidine^24–27^ moieties was observed into several bioactive chemical scaffolds, some of which are anti-inflammatory, antihyperglycemic, or antimycobacterial, etc. A number of structure–activity relationship (SAR) studies, following the molecular hybridisation approach on compounds bearing these motifs was accomplished for designing new anti-inflammatory agents *via* the introduction of different pharmacophoric subunits (Figure 1).^24^^,^^28–31^

As a result of these findings and as part of our continuing project committed to the synthesis of diverse heterocycles as anti-inflammatory agents with better safety profile, a novel series of compounds bearing oxadiazoles, thiadiazoles and pyrimidines linked with antipyrine structure as potential anti-inflammatory medicines was synthesized and described as potential anti-inflammatory medicine agents (Figure 1).^12^^,^^13^^,^^20^^,^^23^^,^^25^

On the basis of the molecular hybrid-pharmacophore strategy, functionalization of the antipyrine amino functional group with different substituted amide moieties was carried out to hybridise two active pharmacophores in one molecular frame. SAR has been investigated and the promising candidates have been computationally investigated to better understand their behaviour inside the potential pocket of COX-2 enzymes.

Anti-inflammatory potential of the target compounds was assessed via different *in vitro* assays (COX-1 and COX-2. On the basis of *in vitro* results, the *in vivo* anti-inflammatory investigations were executed on the highly potent structures using carrageenan paw edema model. The potential underlying mechanisms of **4b** and **4c** were elucidated by investigating their effect on NF-κB, which controls the expression of different pro-inflammatory genes encoding COX-2 and cytokines like TNF-α. Furthermore, the effects on the production level of PGE_2_ as well as the oxidative and nitrosative stress were thoroughly investigated. The selectivity profile of the target structures against cyclooxygenase isoforms was augmented by the virtual docking simulations. Significant interactions between selective COX-2 drugs and the amino acid residues in the extra secondary COX-2 enzyme pocket were observed.

## Results and discussion

### Chemistry

**Scheme 1.**
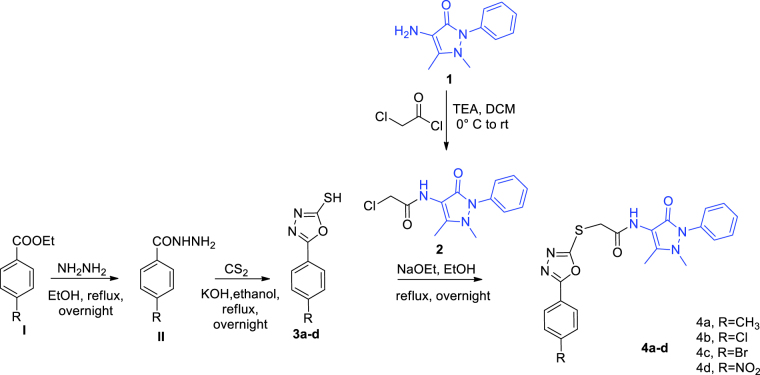
Synthetic pathway of 1,3,4-oxadiazolyl antipyrine derivatives (**4a-d**).

**Scheme 2.**
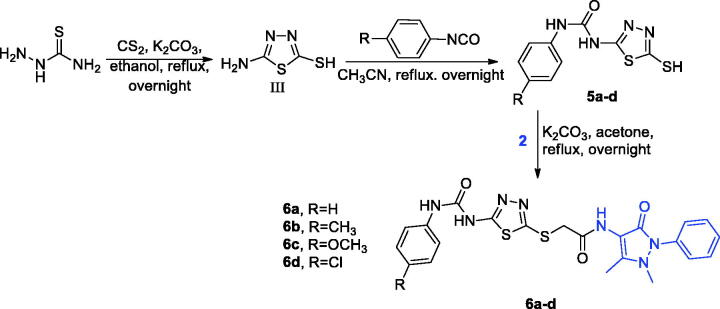
Synthetic pathway of 1,3,4-thiadiazoles derivatives (**6a-d**).

**Scheme 3.**
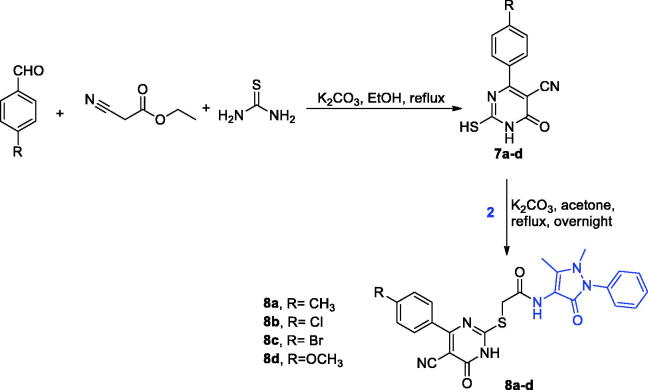
Synthetic pathway of pyrimidines derivatives **8a-d**.

The chemical synthesis of the designed structures, oxadiazoles **4a-d**, thiadiazoles **6a**-**d** and pyrimidines **8a**-**d**, involved many steps which are is illustrated in schemes 1–3. Initially, 4-amino antipyrine **1** was acetylated using chloroacetyl chloride with triethylamine as a base, to furnish 4–(2-chloro acetamido) antipyrine **2**, which in turn has been introduced as key precursor in several nucleophilic substitution reactions.^32^

Oxadiazole-2-thiol derivatives **3a-d** were obtained by the reaction of benzohydrazide derivatives II with carbon disulphide.^33–35^ At the first, 2-mercapto-5-aryl-oxadiazoles **3a-d** were refluxed in acetone with chloroacetamide antipyrine **2** in presence of potassium carbonate as previous reported for alkylation of such thiols, but no conversion was occurred.^33^ Then, NaOEt as strong base was used instead,^36^ led to complete conversion to obtain the corresponding oxadiazole-antipyrine hybrids (**4a-d**; Scheme 1).

By reacting 5-amino-1,3,4-thiadiazole-2-thiol (III) with different phenyl isocyanates under reflux for 5 hours while utilizing acetonitrile as a solvent, the main intermediates 5 were produced **(**Scheme 2).^37^^,^^38^

Also, carbonitrile intermediates **7a-d** were prepared *via* extended heating of various aldehydes with ethyl cyanoacetate and thiourea in ethyl alcohol and K_2_CO_3_ **(**Scheme 3).^39^^,^^40^ At the end, compounds **5a**-**d** and **7a**-**d** were refluxed with chloride **3 2** in acetone in presence of K_2_CO_3_ to obtain our target compounds **6a**-**d** and **8a**-**d**, respectively (Schemes 2 and 3).^39^

The proton-NMR spectra showed a characteristic singlet peak at ∼4.20 ppm, corresponding to -CH_2_- protons confirming the hybrid formation. Furthermore, two characteristic singlet peaks at ∼2.41 (-CH_3_ group) and 3.20 (-NCH_3_ group) are present in all ^1^H-NMR spectra. Furthermore, the IR charts of acid amides **4a**-**d** exhibit two characteristic absorption bands at ∼1704 cm^−1^ and 1670 cm^−1^ due to the presence of two amidic -C = O groups, while **6a-d** and **8a-d** derivatives displayed 3 strong peaks at∼1704 cm^−1^, 1704 cm^−1^ and 1670 cm^−1^ attributed for three -C = O groups stretching vibration.

^13^C-NMR showed the presence of two peaks in derivatives **4a**-**d** or three peaks in derivatives **6a-d** and **8a-d** at ∼160–169 ppm equivalent to the C=O carbons. In addition, all hybrids showed three peaks at aliphatic region at nearly 11–39 ppm that exhibit methyl carbons.

### Anti-inflammatory studies

#### In vitro cyclooxygenase (COX) inhibition assay

The *in vitro* assay was done to test the ability of the new hybrids to cause both COX-1 and COX-2 subtypes inhibition (**Table 1**). The results revealed that **4b-d** and **8d** were the most promising and powerful derivatives in comparison to celecoxib with a half-maximal inhibitory concentration(IC_50_) range of 53–69 nM. Considering COX-2 selectivity index along with potency, compounds **4b** and **4c** possess the highest two selectivity indexes, 4.4 4 and 1.86, respectively, among these potent derivatives. Accordingly, compounds **4b** and **4c** seemed to be the most promising candidates among all the synthesized derivatives.

**Table 1.** IC_50_ values of the tested compounds as COX inhibitors.

	Comp. no.	**COX-2** **(IC_50_ nM)^a^ ± SD**	**COX-1** **(IC_50_ nM)^a^ ± SD**	(SI)^b^
1	4a	86.92 ± 2.19	178.5 ± 2.15	2.05
2	**4b**	53.76 ± 2.05	238.5 ± 2.08	4.44
3	**4c**	69.79 ± 2.14	129.5 ± 2.30	1.86
4	4d	65.54 ± 2.23	50.76 ± 2.18	0.77
5	6a	154.80 ± 2.07	131.60 ± 2.36	0.85
6	6b	103.70 ± 2.25	82.27 ± 2.28	0.79
7	6c	86.10 ± 2.26	282.10 ± 2.34	3.28
8	6d	169.00 ± 2.34	106.00 ± 2.16	0.63
9	8a	88.62 ± 2.19	138.80 ± 2.31	1.57
10	8b	119.80 ± 2.06	209.00 ± 2.21	1.74
11	8c	129.80 ± 2.10	163.60 ± 2.39	1.26
12	8d	68.74 ± 2.19	91.59 ± 2.15	1.33
Celecoxib		45.93 ± 2.19	309.60 ± 2.16	6.74

**^a^**Data are presented as IC_50_ values of three different experiments using the human COXs (1 and 2) assay kit.

^b^*In vitro* COX-2 selectivity index = (COX-1 IC_50_/COX-2 IC_50_).

These results demonstrated the potent anti-inflammatory activity of oxadiazoles antipyrine antipyrines and suggested that introduction of *p*-chloro/bromo group on the phenyl moiety of oxadiazole, as in compounds **4 b** and **4c**, respectively, adds some degree of selectivity towards the inhibition of COX-2.

**Figure 2.**
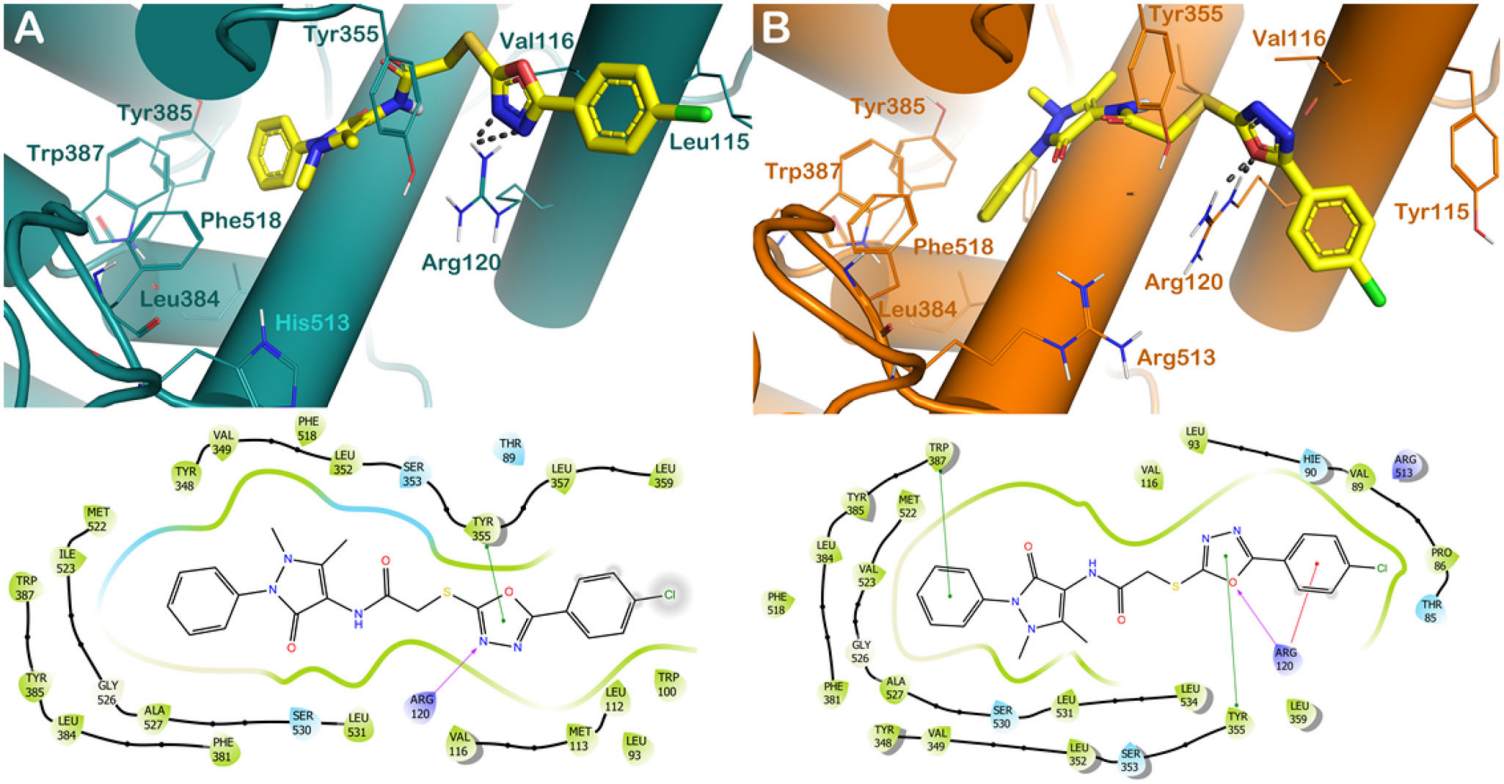
(A) Proposed binding interactions of compound **4b** into COX-1 enzyme (PDB ID 6Y3C). (B) Proposed binding interactions **4b** into COX-2 enzyme (PDB ID 5KIR). Important residues in the active site are indicated by lines, while the gray dotted lines are used for representing H-bonds. For the sake of clarity, nonpolar Hs were removed. In the ligand-interaction diagrams the magenta arrow represents the H-bond, the green line the π–π stacking, the red line the cation-π stacking.

To obtain information on the binding interactions of the two promising compounds, **4b** and **4c**, into COX isoforms, wehave applied virtual molecular docking studies. In general, considering the different degrees of magnitude for the selected compounds in inhibiting COX isoforms, we detected a little drop in the number of contacts when the compounds were docked into COX-1 enzyme with respect to the output obtained for the COX-2 isoform, this is also reflected in a reduction in computational scores. In fact, as reported in Figure 2(A), the compound **4 b** into COX-1 was able to establish an Hydrogen bond with Arg120 and a π–π stacking with Tyr355 by the oxadiazole moiety, while when docked into COX-2, **4 b** increased the number of contacts, establishing, in addition to the H-bond with Arg120 by the oxadiazole moiety, a series of hydrophobic interactions with Tyr355 and Trp387 (π–π stacking), and with Arg120 (cation-π stacking) by the oxadiazole moiety, the benzyl group, and the chlorobenzene ring, respectively (Figure 2(B)). These binding modes accounted for a different docking score of **4 b** into the two enzymes (GlideScore: **4 b**/COX-1 = −7.904 kcal/mol, reference compound celecoxib −8.211 kcal/mol; **4 b**/COX-2 = −9.221 kcal/mol, reference compound celecoxib −9.631 kcal/mol), in agreement with the diverse inhibitory potency against the two enzymes as found by *in vitro* studies.

**Figure 3.**
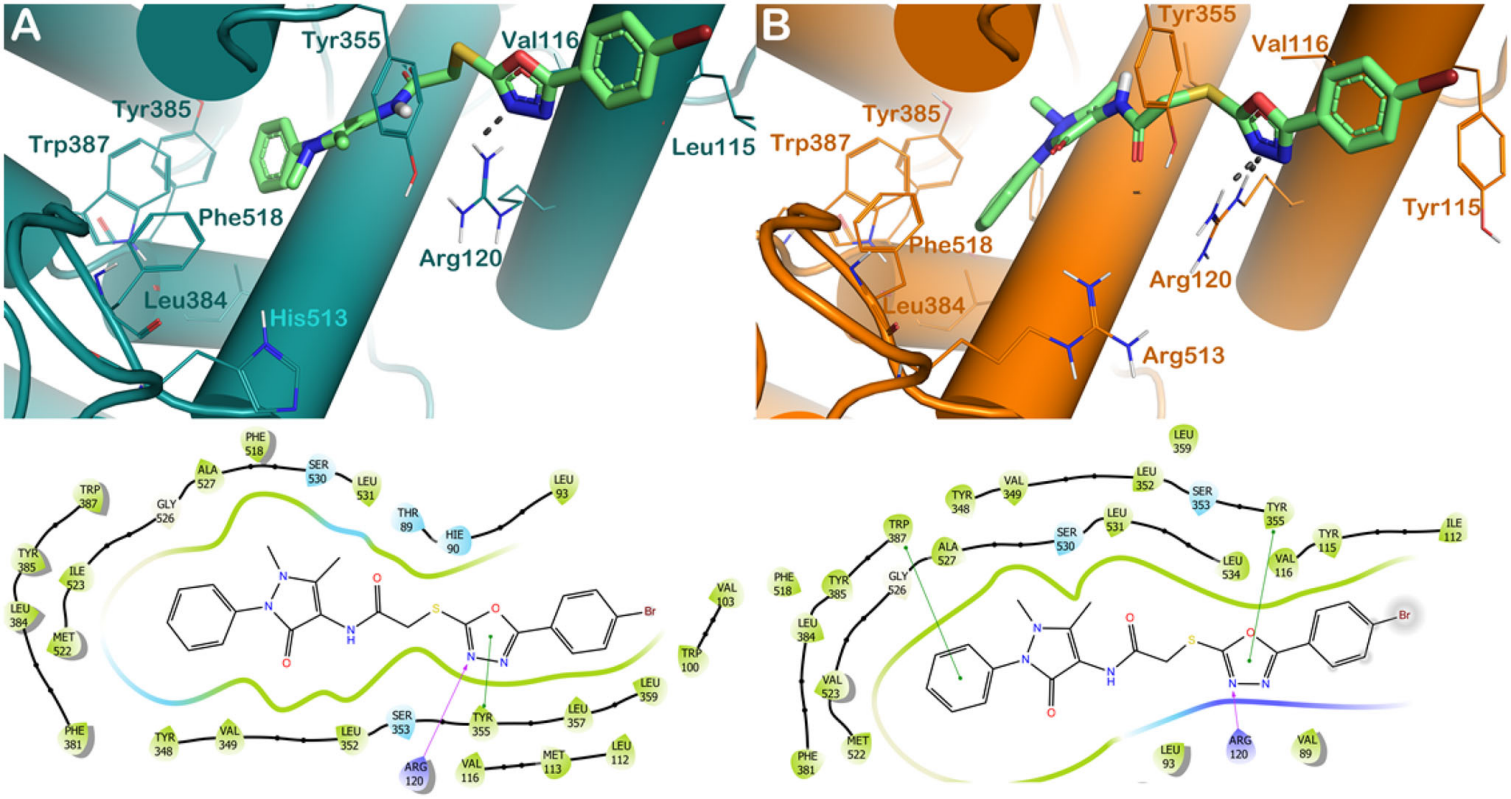
(A) Proposed binding interactions **4c** into COX-1 enzyme (PDB ID 6Y3C). (B) Proposed binding interactions **4c** into COX-2 enzyme. In the ligand-interaction diagrams the magenta arrow represents the H-bond, the green line the π–π stacking, the red line the cation-π stacking.

The same trend was noted for compound **4c**. In effect, this compound is able to produce the same interactions found for **4 b** when it is docked into COX-1 enzyme, targeting Arg120 (H-bond) and Tyr355 (π–π stacking) by the oxadiazole moiety (Figure 3(A)). Considering COX-2, the active site's key residues, we noticed a rise in the number of interactions. Compound **4c**, also in this case, is able to establish a strong network of hydrophobic interactions into COX-2, targeting Tyr355 and Trp387 by a π–π stacking by the oxadiazole moiety and benzyl group, respectively, and Arg120 by a polar contact established by the oxadiazole moiety** (**Figure 3(B)). These binding modes accounted for a different docking score of **4c** into the two enzymes (GlideScore: **4c**/COX-1 = −8.224 kcal/mol; **4c**/COX-2 = −9.014 kcal/mol), again in agreement with the experimental data.

**Figure 4.**
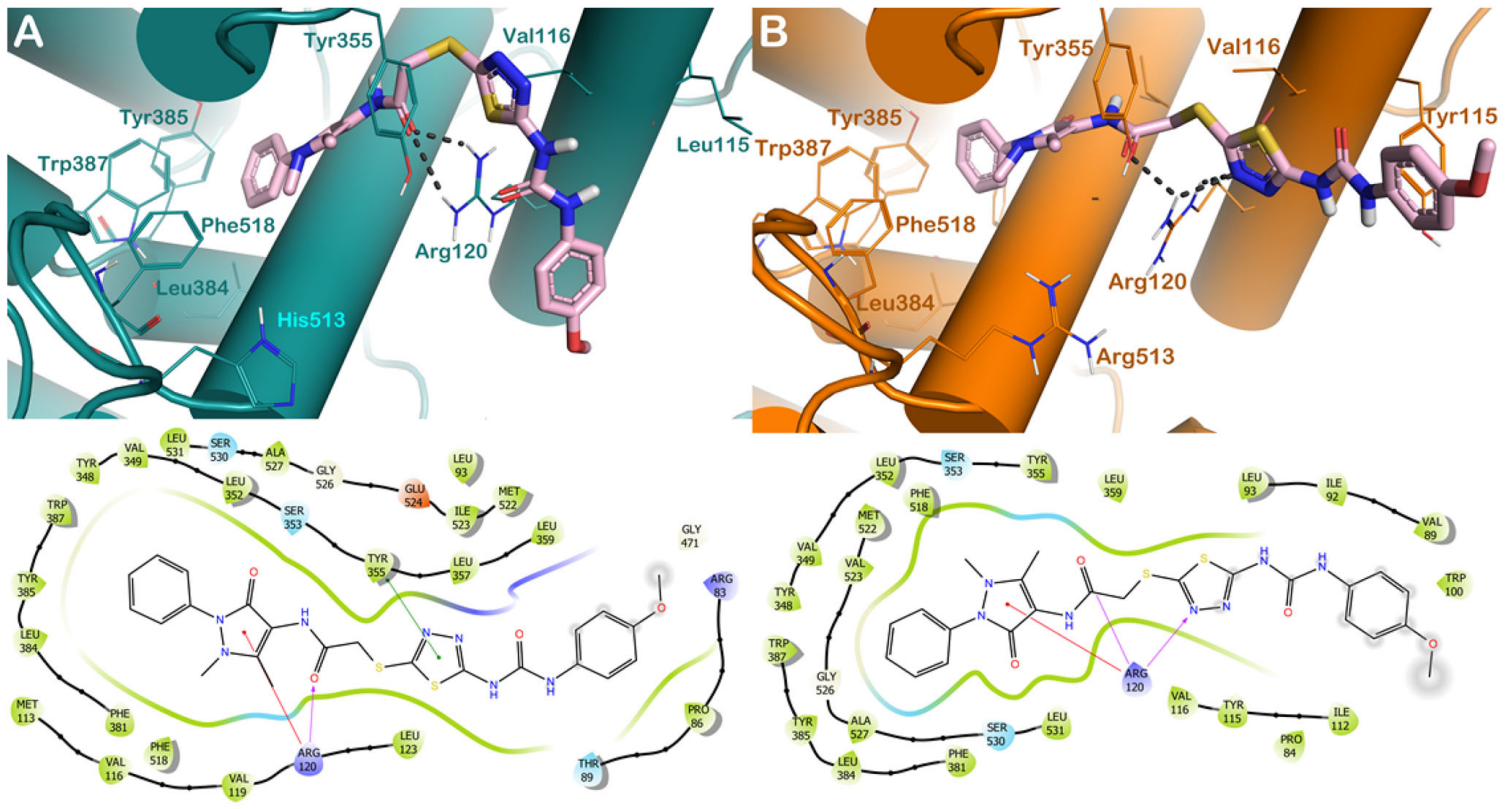
(A) Proposed binding interactions **6c** into COX-1 enzyme (PDB ID 6Y3C). (B) Proposed binding interactions **6c** into COX-2 enzyme. In the ligand-interaction diagrams the magenta arrow represents the H-bond, the green line the π–π stacking, the red line the cation-π stacking.

Considering other members of the series, we evaluated the binding mode of compound **6c**. Based on docking calculations, this compound when docked within the binding site of COX-1 can target Arg120 by H-bond and cation-π stacking by the carbonyl group and the methyl pyrazole moiety, respectively, while we also observed a π–π stacking with Tyr355 by the thiadiazol moiety **(**Figure 4(A)**).** The docking output of **6c** into COX-2 showed that compound **6c** can target Arg120 by several H-bonds established by the carbonyl group and the thiadiazol moiety, and in addition can establish a cation-π stacking by the methyl pyrazole moiety with the same residue. These binding modes accounted for a different docking score of **6c** into the two enzymes (GlideScore: **6c**/COX-1 = −8.372 kcal/mol; **6c**/COX-2 = −9.236 kcal/mol), accounting to the diverse potency of the compound found by the experimental procedures.

**Figure 5.**
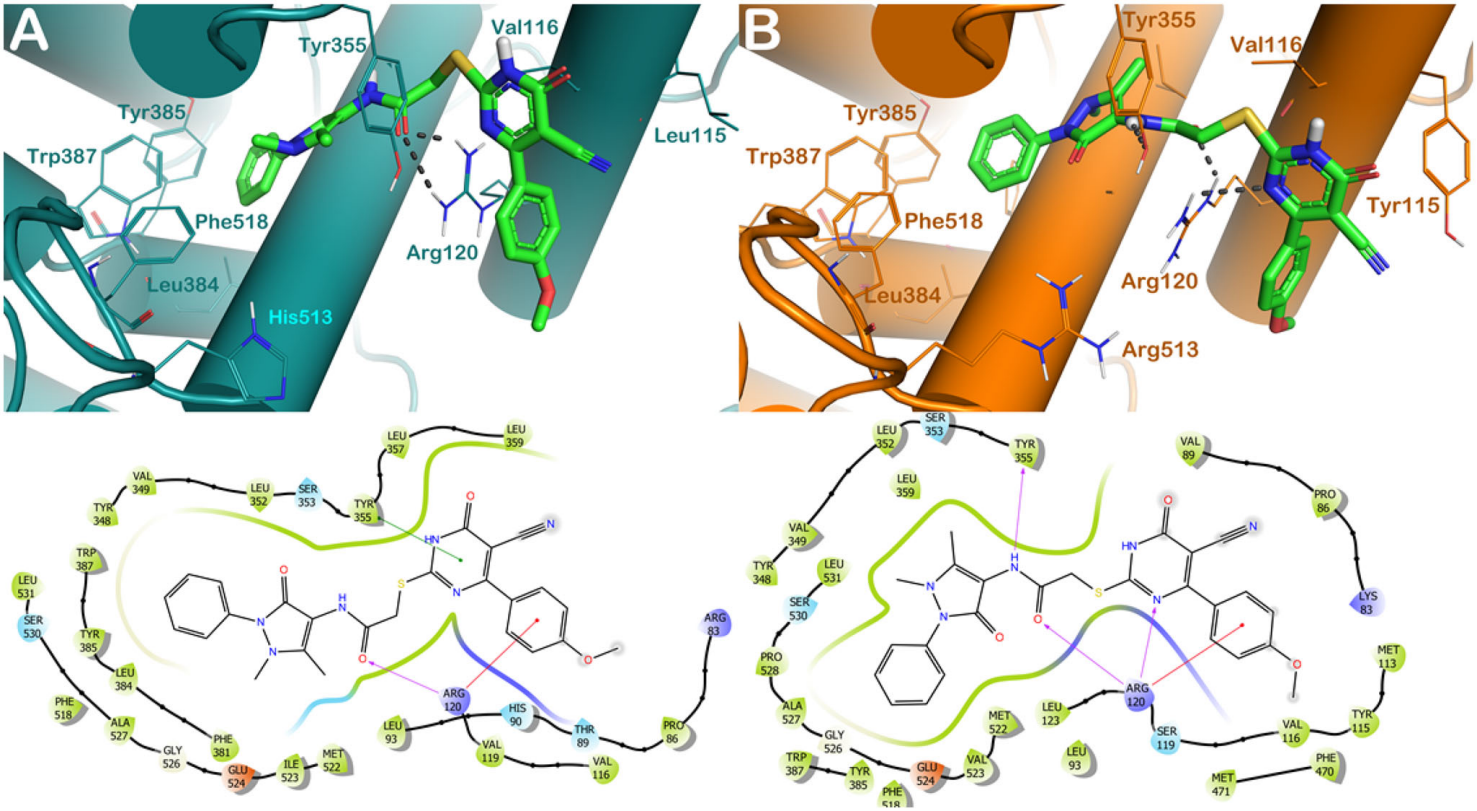
(A) Proposed binding interactions **8d** into COX-1 enzyme (PDB ID 6Y3C). (B) Proposed binding interactions **8d** into COX-2 enzyme. In the ligand-interaction diagrams the magenta arrow represents the H-bond, the green line the π–π stacking, the red line the cation-π stacking.

Considering compound **8d**, we observed favourable interactions within the COX-1 binding site, being able to target the key residues Arg120 by H-bonds by the carbonyl group, while can establish π–π stacking with Tyr355 by the dihydropyrimidine nucleus and a cation-π stacking with Arg 120 by the methoxybenzene moiety (Figure 5(A)). Considering COX-2, we noted a small increase in the number of interactions with the key residues of the active site. Compound 8d can target Arg120 and Tyr355 by H-bonds established by the dihydropyrimidine nucleus, the carbonyl group, and the NH, respectively, and it can establish a cation-π stacking with Arg120 by the methoxybenzene moiety (Figure 5(B)). These binding modes accounted for a similar docking score of **8d** into the two enzymes (GlideScore: **8d**/COX-1 = −9.093 kcal/mol; **8d**/COX-2 = −9.113 kcal/mol), in agreement with the experimental data.

#### Carrageenan-induced rat paw edoema

The *in vivo* anti-inflammatory potential of the selected hybrids resulted from the above COX-2/COX-1 screening, **4 b** and **4c**, were evaluated against rat paw edoema occured by carrageenan, which is a well-documented model of acute inflammation to study the anti-inflammatory activity of synthetic compounds as it involves various inflammatory mediators and has greater reproducibility.

##### Effects on paw edoema and edoema inhibition rate

**Figure 6.**
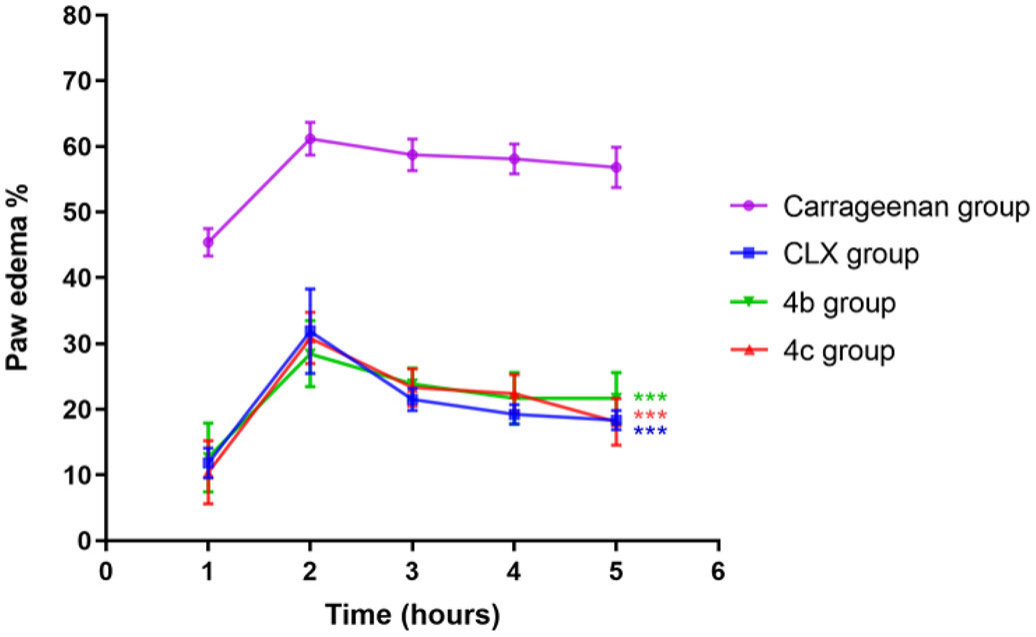
Effects of celecoxib (CLX), **4 b**, and **4c** on carrageenan-induced paw edoema. *** *p* < 0.001 compared to the carrageenan group.

Injection of carrageenan-induced paw swelling in a time-dependent manner (Figure 6) where an increase in the percentage of paw swelling reached a maximum after 2 h followed by minor decreases till became near constant at the final stage of the experiment (5 h). Pre-treatment with celecoxib (CLX), **4b**, and **4c** resulted in the same trend but as expected, with marked lower percentages of paw swelling compared to the carrageenan group. These results demonestrated the promising anti-inflammatory potential of **4b**, and **4c**, compared to CLX in the model which would be further confirmed via histopathological evaluation of the inflamed tissue.

##### Histopathological evaluation of inflamed tissue

**Figure 7.**
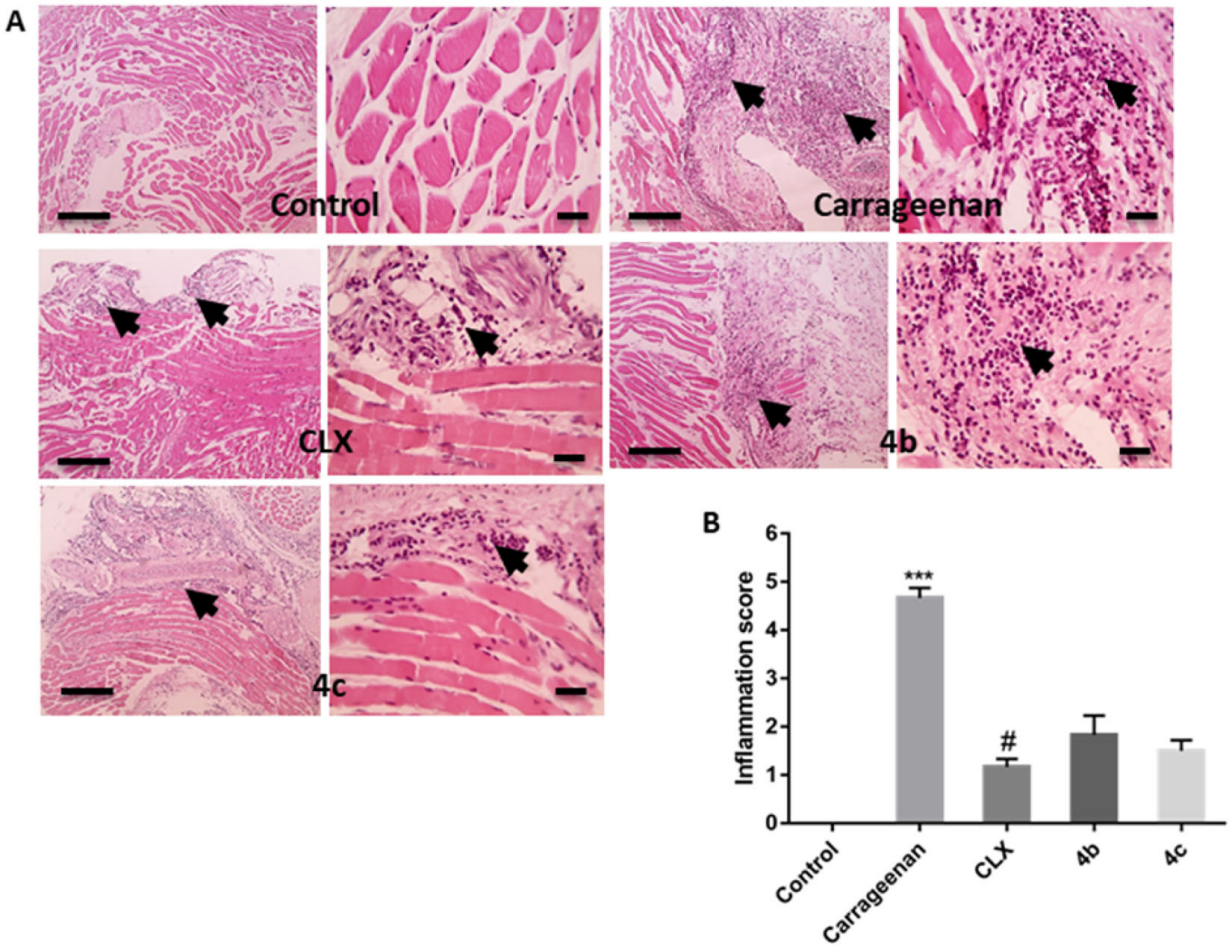
(A) Representative images of hematoxylin-eosin-stained paw sections showing areas of inflammation (arrows). (B) Semi-quantitative scoring of inflammation. *** *p* < 0.001 compared to the control group; # *p* < 0.05 compared to the carrageenan group.

Carrageenan-injected rat paw tissues showed severe inflammation characterised by enormous neutrophil infiltration. Interestingly, pre-treatment with not only CLX, but also **4 b**, and **4c** markedly reduced such inflammatory reaction (Figure 7(A)). Semi-quantitative determination of inflammation scores demonstrated a significant (*P* < 0.001) increase in the of the carrageenan group compared to the control one. This score was decreased upon pre-treatment with CLX, **4 b**, and **4c** and the difference between the three treated groups was statistically significant. However, this decrease was statistically significant (*P* < 0.05) only with the CLX group in comparison with the carrageenan group (Figure 7(B)).

##### Effects on NF-κB activation and COX-2 expression level

**Figure 8.**
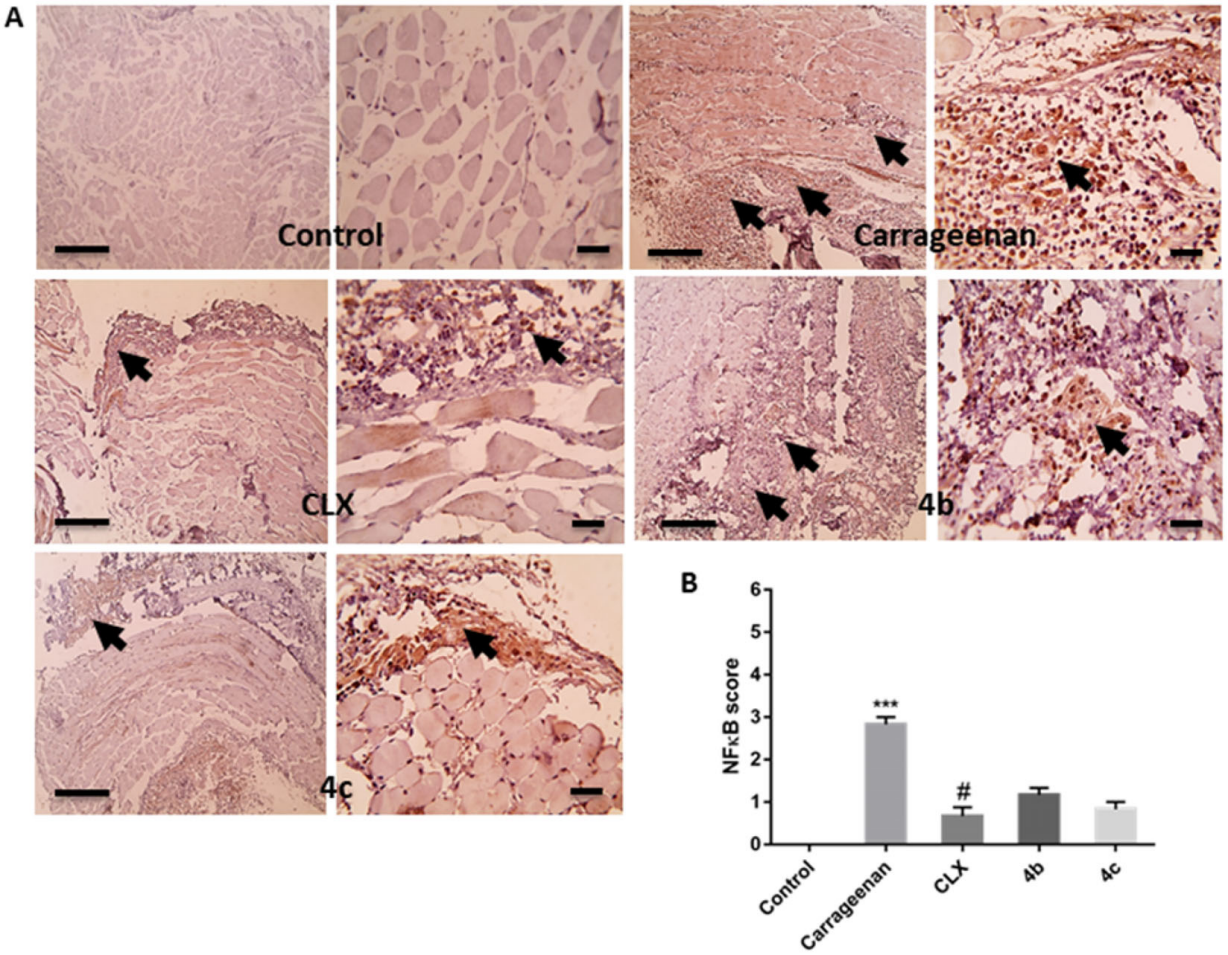
(A) Representative images of immunostained paw sections showing NF-κB positive expression (arrows). (B) Semi-quantitative scoring of NF-κB positive expression. *** *p* < 0.001 in comparison to the control group; # *p* < 0.05 compared to the carrageenan group.

**Figure 9.**
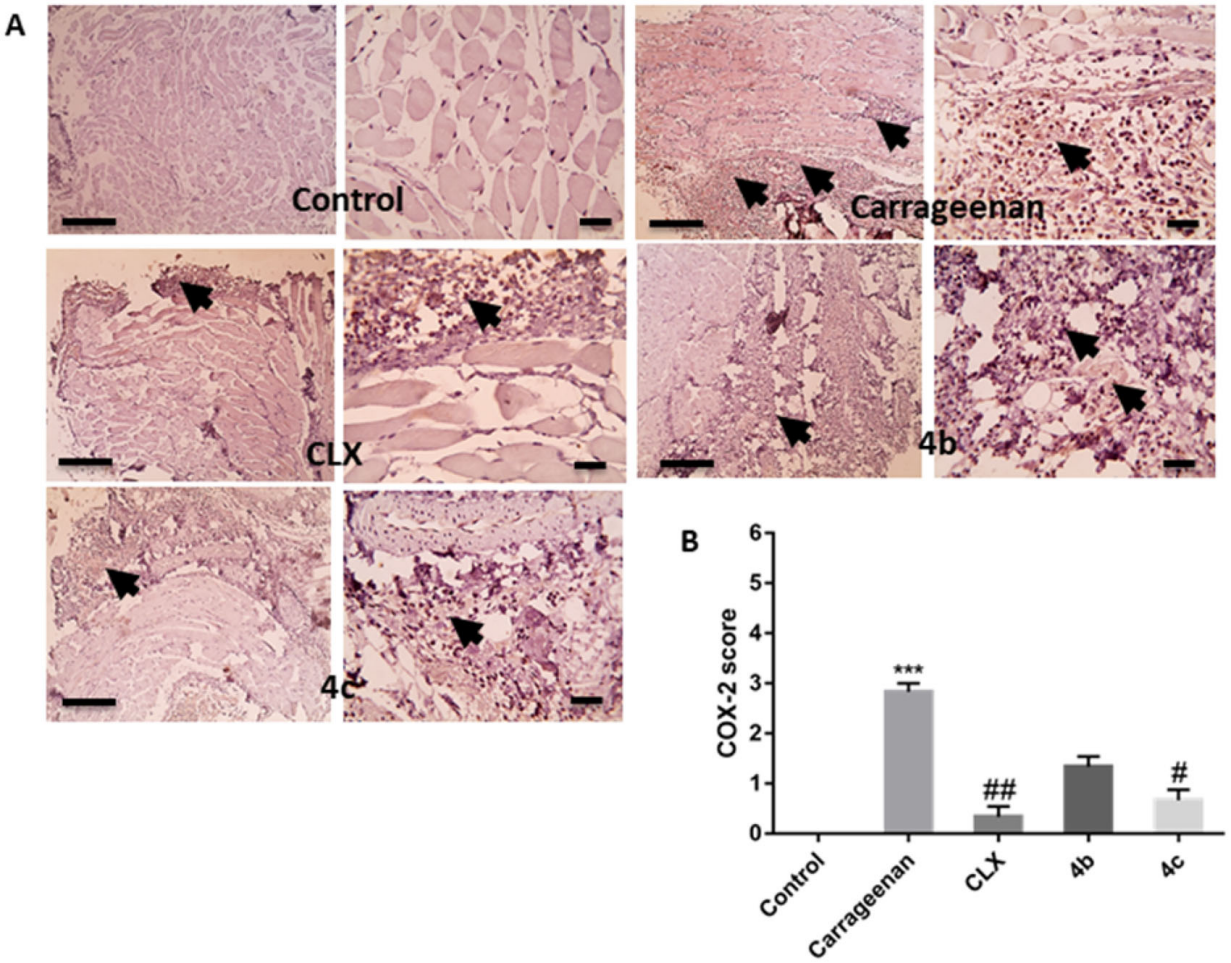
(A) Representative images of immunostained paw sections showing COX-2 positive expression (arrows). (B) Semi-quantitative scoring of COX-2 positive expression. *** *p* < 0.001 compared to the control group; # *p* < 0.05, ## *P* < 0.01 compared to the carrageenan group.

The transcription factor NF-κB plays a vital role in mediating inflammatory responses. Upon activation and translocation to the nucleus, it mediates the expression of different pro-inflammatory genes such as those encoding inducible enzymes like COX-2 and cytokines like TNF-α.^41^ COX-2 represents a crucial enzyme in inflammatory reactions as it is known to be the rate-limiting enzyme, which induces the production of pro-inflammatory PGs, mainly PGE2, at the site of inflammation.^42^ Herein, the immunohistochemistry results showed that pre-treatment with CLX, **4 b**, and **4c** suppressed the marked elevation of the immuno-expression of NF-κB (Figure 8(A)) and COX-2 (Figure 9(A)) induced by carrageenan in paw tissue. Furthermore, semi-quantitative scoring of NF-κB (Figure 8(B)) and COX-2 (Figure 9(B)) positive expression illustrated that carrageenan significantly (*P* < 0.001) increased their expression score in comparison with the control group, meanwhile pre-treatment with CLX, **4 b**, and **4c**, decreased their expression score and the difference between the threetreated groups was insignificant. However, this decrease in NF-κB score was only statistically significant (*P* < 0.05) only with the CLX group when compared to the carrageenan group.

##### Effects on TNF-α and PGE2 tissue levels

**Figure 10.**
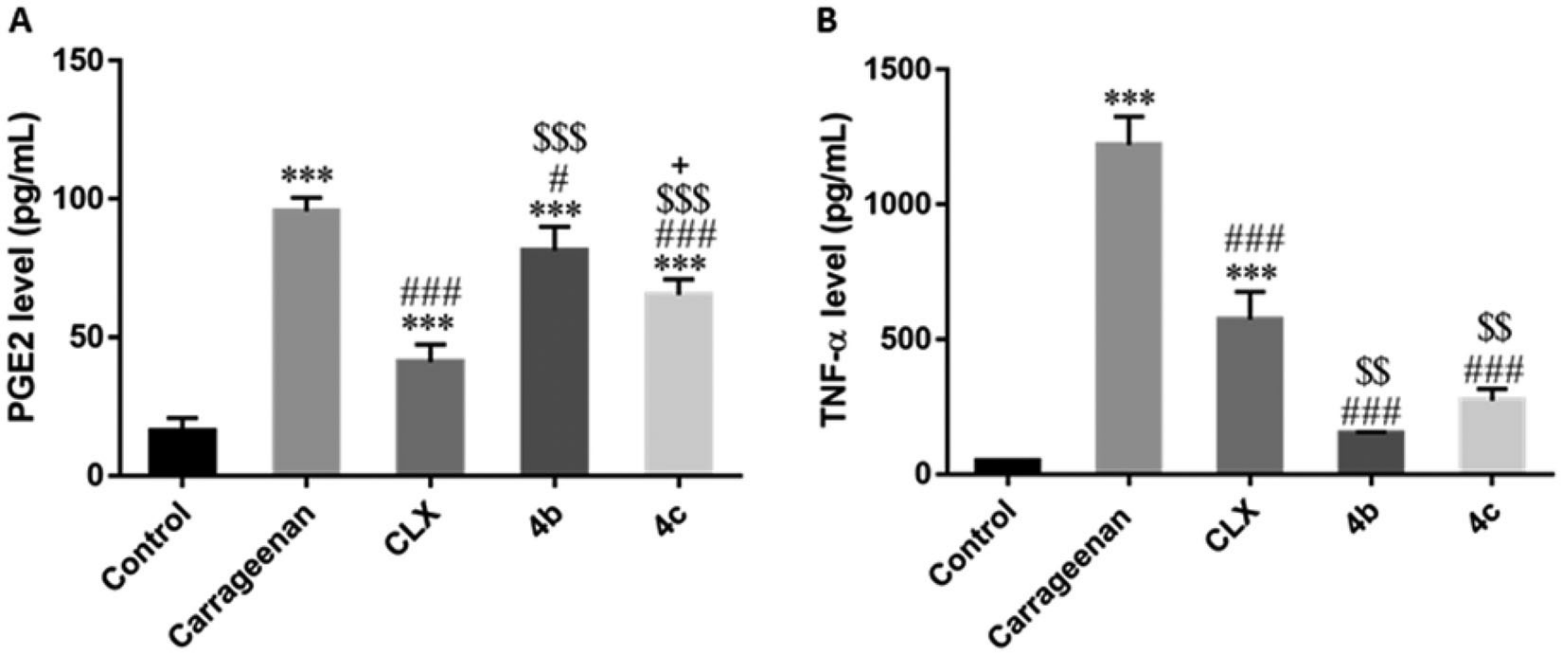
Effects of Celecoxib (CLX), **4 b**, and **4c** on carrageenan-induced elevation of (**A)** Prostaglandin E2 (PGE2) and (**B)** Tumour necrosis factor-alpha (TNF-α) paw tissue levels. *** *p* < 0.001 compared to the control group; # *p* < 0.05, ### *P* < 0.001 compared to the carrageenan group; $$*p* < 0.01, $$$*P* < 0.001 compared to the CLX group; + *p* < 0.05 compared to the **4 b** group.

PGE2 is the principal mediator of inflammatory disorders and still the principal target for anti-inflammatory therapy.^43^ In the present study, PGE2 level was significantly (*P* < 0.001) increased by carrageenan injection compared to the control group. Pre-treatment with CLX, **4 b**, and **4c** significantly (*P* < 0.001, 0.05, and 0.001, respectively) reduced PGE2 level in comparison to the carrageenan group. However, both **4 b** and **4c** groups showed significantly (*P* < 0.001) higher levels in comparison to the CLX group. Of note, PGE2 level was significantly (*P* < 0.05) lower in the **4c** compared to the **4b** groups (Figure 10(A)).

TNF-α is a potent pro-inflammatory cytokine that amplifies almost all acute inflammatory reactions.^44^ Our results showed that carrageenan injection significantly (*P* < 0.001) increased TNF-α level. Upon pre-treatment either with CLX, **4 b**, or **4c**, there was a significant (*P* < 0.001) decrease in the TNF-α level compared to the carrageenan group. Interestingly, this effect was significantly (*P* < 0.01) higher with both **4 b** and **4c** than CLX (Figure 10(B)).

##### Effects on oxidative and nitrosative stress

**Figure 11.**
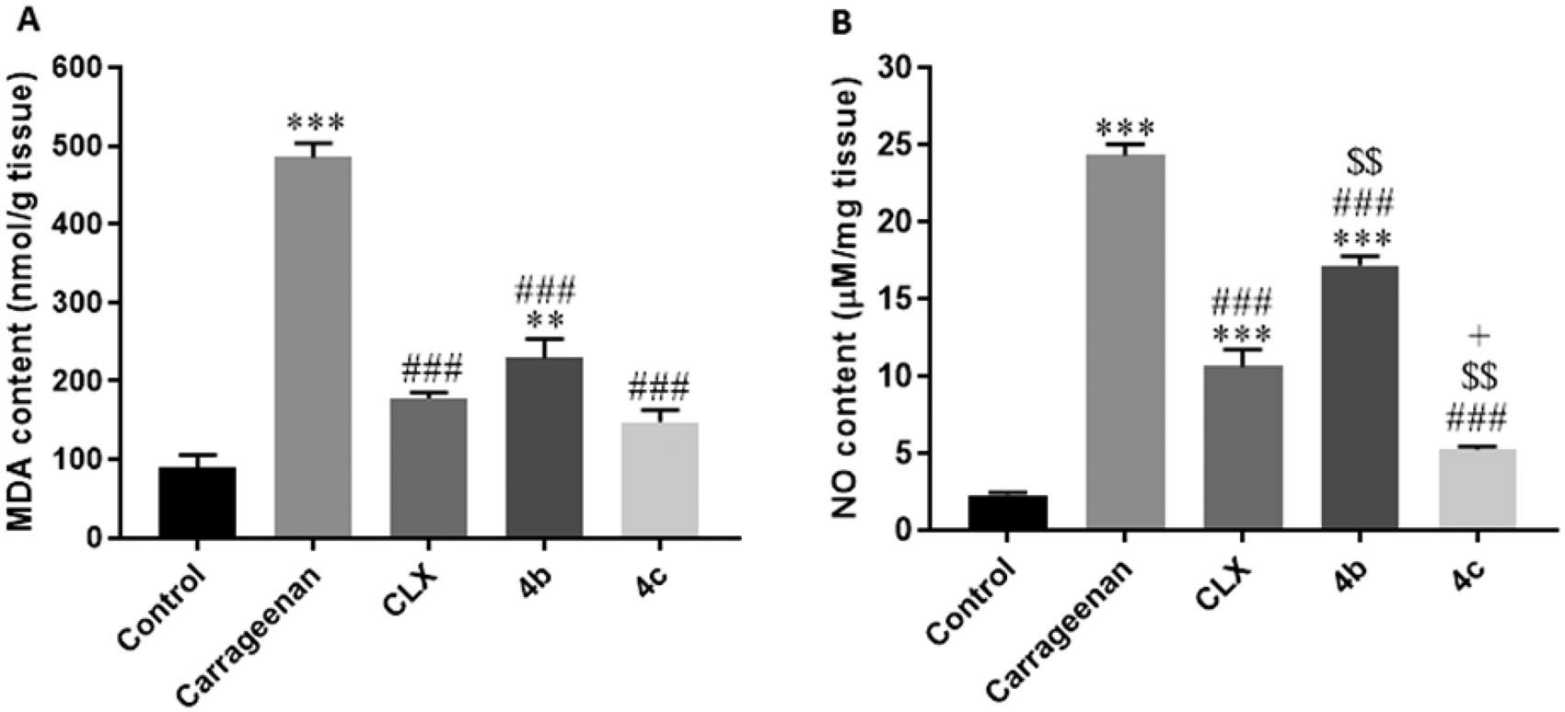
Effects of Celecoxib (CLX), **4 b**, and **4c** on carrageenan-induced elevation of (A) malondialdehyde (MDA) and (B) Nitric oxide (NO) paw tissue contents. ** *p* < 0.01, *** *p* < 0.001 compared to the control group; # *p* < 0.05, ### *P* < 0.001 compared to the carrageenan group; $$*p* < 0.01 compared to the CLX group; + *p* < 0.05 compared to the **4 b** group.

Oxidative stress is closely connected to inflammatory reactions. Reactive oxygen species (ROS) may modulate NF-κB signals to produce pro-inflammatory cytokines like TNF-α. Furthermore, oxidative stress has been reported to be linked to COX-2 induction with subsequent PGs production.^45^^,^^46^ Additionally, an association has been evidenced between NO, a key mediator of acute inflammation, and COX activity, where NO activates COX enzymes enhancing PGs synthesis.^47^ In the current study, carrageenan injection induced a status of both oxidative and nitrosative stress indicated by significantly (*P* < 0.001) elevated in paw tissue contents of the lipid peroxidation product, malondialdehyde (MDA) (Figure 11(A)) and the nitrosative stress product, NO (Figure 11(B)), respectively in comparison with the control group. However, pre-treatment with CLX, 4 b, and 4c significantly (*P* < 0.001) reduced both levels compared to the control group. Notably, the difference between the three treated groups was significant regarding the NO content. Interestingly, the content was significantly (*P* < 0.01 and 0.05, respectively) lower with **4c** compared to CLX and **4 b**, and it was significantly (*P* < 0.01) higher with **4 b** than with CLX.

## Conclusion

Following the synthetic methods described in the literature, a novel series of antipyrine-based scaffolds was designed and synthesised by combining pyrimidines, 1,3,4-oxadiazoles, and 1,3,4-thiadiazoles motifs. A variety of spectroscopic methods were used to confirm the chemical structures of the synthesised compounds. The carrageenan-induced paw edoema model and other *in vitro* assays were applied to evaluate the target compounds as potential anti-inflammatory agents. Additionally, the selective inhibitory activity towards COX-2 enzymes was assessed, and the results revealed that compounds **4 b** and **4c** represented the most active compounds among the series compounds. The obtained *in vitro* results have been validated using molecular modelling approach. The findings can be applied for the optimisation of the most active compounds in order to get more potent agents with better safety profiles in the near future.

## Experimental

### Chemistry

All specifications regarding melting point measurements, FT-IR 200 spectrophotometer and NMR spectrometry, mass analyser and elemental analysis are previously reported in our published article. The key intermediate **2** and intermediate compounds **5a**-**d**, **7a**-**d** were synthesized according to the previously reported methods in literature.^32–35^^,^^37–40^

#### General procedure for the preparation of 1,3,4-oxadiazole derivatives (4a-d)

To a solution of thiol **3a-d** (0.3 mmol) and NaOEt (2 eq.), stirred for 2 h in ethyl alcohol, was added chloride **2** (0.3 mmol). The mixture was then left to reflux overnight. After the reaction was completed, it was transferred into ice, the precepitate was filtered and purified via recrystallization from ethyl alcohol.

*N*-(1,5-Dimethyl-3-oxo-2-phenyl-2,3-dihydro-1*H*-pyrazol-4-yl)-2-((5-(*p*-tolyl)-1,3,4-oxadiazol-2-yl)thio)acetamide (**4a**)

White coloured powder; (0.08 g, 61%). M.p. 234–236 °C. IR (νmax/cm^−1^): 3451(NH), 1680, 1644 (C = Os). Proton NMR (DMSO-*d_6_*) *δ* 9.63 (s, 1H), 7.89 (d, *J* = 8.0 Hz, 2H), 7.55 − 7.49 (m, 2H), 7.52 (d, *J* = 8.0 Hz, 2H), 7.40 − 7.30 (m, 3H), 4.31 (s, 2H), 3.06 (s, 3H), 2.40 (s, 3H), 2.10 (s, 3H). Carbon-13 NMR (DMSO-*d_6_*) *δ* 166.2, 165.7, 163.4, 161.9, 152.7, 142.7, 135.4, 130.5, 129.6, 126.9, 126.8, 124.1, 120.7, 107.3, 36.8, 36.1, 21.6, 11.6. MS m/z (%): 435.31 (M^+^, 24.33). Anal. Calcd (435.50): C, 60.67; H, 4.86; N, 16.08; S, 7.36. Found: C, 60.78; H, 4.88; N, 16.18; S, 7.28%.

2-((5–(4-Chlorophenyl)-1,3,4-oxadiazol-2-yl)thio)-*N*-(1,5-dimethyl-3-oxo-2-phenyl-2,3-dihydro-1*H*-pyrazol-4-yl)acetamide (**4 b**)

Yellow coloured powder; (0.089 g, 65%). M.p. 190–192 °C. IR (νmax/cm^−1^): 3319 (NH), 1677, 1643 (C = Os). Proton NMR (DMSO-*d_6_*) *δ* 11.02 (s, 1H), 7.85 (d, *J* = 8.4 Hz, 2H), 7.60 − 7.50 (m, 4H), 7.40 − 7.35 (m, 3H), 4.25 (s, 2H), 3.24 (s, 3H), 2.27 (s, 3H). Carbon-13 NMR (DMSO-*d_6_*) *δ* 171.3, 162.7, 162.6, 160.5, 154.3, 136.7, 134.9, 132.6, 130.2, 129.7, 129.0, 127.5, 124.9, 103.4, 35.7, 32.7, 11.0. MS m/z (%): 455.06 (M^+^, 13.51). Anal. Calcd (455.92): C, 55.32; H, 3.98; Cl, 7.78; N, 15.36; S, 7.03. Found: C, 55.14; H, 3.85; Cl, 7.70; N, 15.39; S, 7.09%.

2-((5–(4-Bromophenyl)-1,3,4-oxadiazol-2-yl)thio)-*N*-(1,5-dimethyl-3-oxo-2-phenyl-2,3-dihydro-1*H*-pyrazol-4-yl)acetamide (**4c**)

Yellow coloured powder; (0.089 g, 59%). M.p. 173–175 °C. IR (νmax/cm^−1^): 3443 (NH), 1735, 1659 (C = Os). Proton NMR (DMSO-*d_6_*) *δ* 10.93 (s, 1H), 7.81 (d, *J* = 7.9 Hz, 2H), 7.68 (d, *J* = 7.9 Hz, 2H), 7.62–7.50 (m, 2H), 7.43–7.35 (m, 3H), 4.21 (s, 2H), 3.24 (s, 3H), 2.26 (s, 3H). Carbon-13 NMR (DMSO-*d_6_*) *δ* 171.4, 163.0, 161.2, 160.5, 153.9, 134.8, 133.9, 131.9, 129.9, 129.7, 127.6, 125.2, 125.1, 103.4, 35.6, 32.5, 11.1. MS m/z (%): 500.4 (M^+^, 22.56). Anal. Calcd (500.37): C, 50.41; H, 3.63; Br, 15.97; N, 14.00; S, 6.41. Found: C, 50.30; H, 3.55; Br, 16.04; N, 14.15; S, 6.23%.

*N*-(1,5-Dimethyl-3-oxo-2-phenyl-2,3-dihydro-1*H*-pyrazol-4-yl)-2-((5–(4-nitrophenyl)-1,3,4-oxadiazol-2-yl)thio)acetamide (**4d**)

Yellow coloured powder; (0.076 g, 54%). M.p. 214–216 °C. IR (νmax/cm^−1^): 3446 (NH), 1737, 1656 (C = Os). Proton NMR (DMSO-*d_6_*) *δ* 11.36 (s, 1H), 8.33 (d, *J* = 8.2 Hz, 2H), 8.07 (d, *J* = 8.2 Hz, 2H), 7.55 (t, *J* = 7.3 Hz, 2H), 7.39 (d, *J* = 7.3 Hz, 3H), 4.24 (s, 2H), 3.25 (s, 3H), 2.27 (s, 3H). Carbon-13 NMR (DMSO-*d_6_*) *δ* 171.4, 162.5, 162.0, 160.5, 154.2, 149.5, 139.9, 134.9, 129.7, 129.3, 127.5, 125.0, 124.1, 103.3, 35.7, 32.7, 11.0. MS m/z (%): 466.10 (M^+^, 33.12). Anal. Calcd (466.47): C, 54.07; H, 3.89; N, 18.02; S, 6.87. Found: C, 54.07; H, 3.90; N, 17.90; S, 6.82%.

#### General procedure for the preparation of 1,3,4-thiadiazole derivatives (6a-d)

The suitable thiol hybrids **5a-d** (0.3 mmole) with K_2_CO_3_ (0.45 mmole) in acetone were allowed to be stirred at rt for half an hour followed by addition of equivalent amount of **2. **The obtained mixture was left for reflux overnight.After that, the reaction was poured into ice, the solid was filtered off and recrystallized from ethyl alcohol to give the target compounds.

*N*-(1,5-Dimethyl-3-oxo-2-phenyl-2,3-dihydro-1*H*-pyrazol-4-yl)-2-((5–(3-phenylureido)-1,3,4-thiadiazol-2-yl)thio)acetamide (**6a**)

Pale yellow coloured powder; (0.097 g, 65%). M.p. 254–256 °C. IR (νmax/cm^−1^): 3451, 3353, 3244 (NHs), 1716, 1671, 1638 (C = Os). Proton NMR (DMSO-*d_6_*) *δ* 11.07 (s, 1H), 9.51 (s, 1H), 9.08 (s, 1H), 7.50 (d, *J* = 6.9 Hz, 4H), 7.44–7.31 (m, 5H), 7.08 (d, *J* = 6.9 Hz, 1H), 4.13 (s, 2H), 3.06 (s, 3H), 2.12 (s, 3H). Carbon-13 NMR (DMSO-*d_6_*) *δ* 166.8, 162.0, 161.3, 157.4, 152.7, 138.8, 135.4, 129.6, 129.4, 126.8, 124.1, 123.6, 119.4, 107.4, 37.6, 36.4, 11.6. MS m/z (%): 495.33 (M^+^, 32.55). Anal. Calcd (495.58): C, 53.32; H, 4.27; N, 19.78; S, 12.94. Found: C, 53.19; H, 4.20; N, 19.80; S, 12.99%.

*N*-(1,5-Dimethyl-3-oxo-2-phenyl-2,3-dihydro-1*H*-pyrazol-4-yl)-2-((5–(3-(*p*-tolyl)ureido)-1,3,4-thiadiazol-2-yl)thio)acetamide (**6 b**)

Pale yellow coloured powder; (0.093 g, 61%). M.p. 253–255 °C. IR (νmax/cm^−1^): 3449, 3352, 3245 (NHs), 1712, 1672, 1638 (C = Os). Proton NMR (DMSO-*d_6_*) *δ* 11.10 (s, 1H), 9.49 (s, 1H), 9.08 (s, 1H), 7.51 (d, *J* = 7.5 Hz, 2H), 7.41–7.30 (m, 5H), 7.15 (d, *J* = 7.5 Hz, 2H), 4.12 (s, 2H), 3.06 (s, 3H), 2.27 (s, 3H), 2.11 (s, 3H). Carbon-13 NMR (DMSO-*d_6_*) *δ* 166.9, 162.0, 161.1, 152.7, 151.6, 136.1, 135.4, 132.7, 132.6, 129.8, 129.6, 126.9, 124.1, 119.5, 107.4, 37.5, 36.3, 20.9, 11.6. MS m/z (%): 509.10 (M^+^, 41.13). Anal. Calcd (509.60): C, 54.21; H, 4.55; N, 19.24; S, 12.58. Found: C, 54.05; H, 4.56; N, 19.33; S, 12.42%.

*N*-(1,5-Dimethyl-3-oxo-2-phenyl-2,3-dihydro-1*H*-pyrazol-4-yl)-2-((5–(3-(4-methoxyphenyl)ureido)-1,3,4-thiadiazol-2-yl)thio)acetamide (**6c**)

Pale yellow coloured powder; (0.104 g, 66%). M.p. 250–252 °C. IR (νmax/cm^−1^): 3449, 3348, 3247 (NHs), 1710, 1673, 1637 (C = Os). Proton NMR (DMSO-*d_6_*) *δ* 11.05 (s, 1H), 9.50 (s, 1H), 8.94 (s, 1H), 7.51 (d, *J* = 7.6 Hz, 2H), 7.44–7.31 (m, 5H), 6.91 (d, *J* = 7.6 Hz, 2H), 4.12 (s, 2H), 3.73 (s, 3H), 3.05 (s, 3H), 2.11 (s, 3H). Carbon-13 NMR (DMSO-*d_6_*) *δ* 166.9, 162.0, 161.4, 155.8, 152.7, 135.3, 135.1, 131.6, 129.7, 129.6, 126.9, 124.2, 121.3, 114.6, 107.3, 55.7, 37.5, 36.3, 11.6. MS m/z (%): 525.02 (M^+^, 34.07). Anal. Calcd (525.60): C, 52.56; H, 4.41; N, 18.65; S, 12.20. Found: C, 52.50; H, 4.44; N, 18.69; S, 12.35%.

2-((5–(3-(4-Chlorophenyl)ureido)-1,3,4-thiadiazol-2-yl)thio)-*N*-(1,5-dimethyl-3-oxo-2-phenyl-2,3-dihydro-1*H*-pyrazol-4-yl)acetamide (**6d**)

Pale yellow coloured powder; (0.095 g, 60%). M.p. 248–250 °C. IR (νmax/cm^−1^): 3451, 3345, 3246 (NHs), 1712, 1672, 1637 (C = Os). Proton NMR (DMSO-*d_6_*) *δ* 11.29 (s, 1H), 9.49 (s, 1H), 8.90 (s, 1H), 7.57 − 7.50 (m, 4H), 7.41 − 7.31 (m, 5H), 4.13 (s, 2H), 3.05 (s, 3H), 2.10 (s, 3H). Carbon-13 NMR (DMSO-*d_6_*) *δ* 166.8, 162.0, 161.3, 157.4, 152.7, 137.5, 135.4, 133.3, 129.4, 126.8, 124.1, 121.6, 119.4, 107.4, 37.6, 36.4, 11.6. MS m/z (%): 529.89 (M^+^, 25.23). Anal. Calcd (530.02): C, 49.85; H, 3.80; Cl, 6.69; N, 18.50; S, 12.10. Found: C, 49.76; H, 3.80; Cl, 6.62; N, 18.55; S, 12.21%.

#### General procedure for the preparation of pyrimidine derivatives (8a-d)

A mixture of thiol intermediates **7a-d** (0.3 mmole) and K_2_CO_3_ (0.45 mmole) was stirred at rt in acetone for half an hour. Then, compound **2** was added. The obtained mixture was heate under reflux overnight. The solid obtained was filtered and dried to afford the target compound.

2-((5-Cyano-6-oxo-4-(*p*-tolyl)-1,6-dihydropyrimidin-2-yl)thio)-*N*-(1,5-dimethyl-3-oxo-2-phenyl-2,3-dihydro-1*H*-pyrazol-4-yl)acetamide (**8a**)

White coloured powder; (0.102 g, 70%). M.p. >300 °C. IR (νmax/cm^−1^): 3451, 3253 (NHs), 2210 (CN), 1687, 1653 (C = Os). Proton NMR (DMSO-*d_6_*) *δ* 9.42 (s, 1H), 7.71 (d, *J* = 8.1 Hz, 2H), 7.51 (t, *J* = 7.8 Hz, 2H), 7.40–7.32 (m, 3H), 7.27 (d, *J* = 8.1 Hz, 2H), 3.85 (s, 2H), 3.03 (s, 3H), 2.36 (s, 3H), 2.05 (s, 3H). Carbon-13 NMR (DMSO-*d_6_*) *δ* 171.9, 171.3, 169.1, 167.7, 162.1, 153.5, 140.3, 135.2, 134.9, 129.6, 129.2, 128.6, 127.0, 124.3, 120.4, 107.3, 89.2, 36.2, 25.6, 21.4, 11.2. MS m/z (%): 486.50 (M^+^, 30.53). Anal. Calcd (486.55): C, 61.71; H, 4.56; N, 17.27; S, 6.59. Found: C, 61.77; H, 4.58; N, 17.20; S, 6.58%.

2-((4–(4-Chlorophenyl)-5-cyano-6-oxo-1,6-dihydropyrimidin-2-yl)thio)-*N*-(1,5-dimethyl-3-oxo-2-phenyl-2,3-dihydro-1*H*-pyrazol-4-yl)acetamide (**8b**)

White coloured powder; (0.114 g, 75%). M.p. 239–241 °C. IR (νmax/cm^−1^): 3448, 3222 (NHs), 2211 (CN), 1687, 1654 (C = Os). Proton NMR (DMSO-*d_6_*) *δ* 9.39 (s, 1H), 7.84 (d, *J* = 8.5 Hz, 2H), 7.54 (d, *J* = 8.5 Hz, 2H), 7.55 − 7.48 (m, 2H), 7.41 − 7.30 (m, 3H), 3.87 (s, 2H), 3.03 (s, 3H), 2.05 (s, 3H). Carbon-13 NMR (DMSO-*d_6_*) *δ* 172.0, 170.5, 168.7, 166.2, 162.1, 152.9, 136.8, 135.5, 135.0, 130.5, 129.6, 128.7, 126.7, 124.0, 120.3, 107.9, 89.5, 36.4, 34.5, 11.5. MS m/z (%): 506.53 (M^+^, 32.73). Anal. Calcd (506.96): C, 56.86; H, 3.78; Cl, 6.99; N, 16.58; S, 6.32. Found: C, 56.76; H, 3.66; Cl, 6.80; N, 16.78; S, 6.30%.

2-((4–(4-Bromophenyl)-5-cyano-6-oxo-1,6-dihydropyrimidin-2-yl)thio)-*N*-(1,5-dimethyl-3-oxo-2-phenyl-2,3-dihydro-1*H*-pyrazol-4-yl)acetamide (**8c**)

White coloured powder; (0.121 g, 73%). M.p. 243–245 °C. IR (νmax/cm^−1^): 3450, 3214 (NHs), 2212 (CN), 1685, 1655 (C = Os). Proton NMR (DMSO-*d_6_*) *δ* 9.39 (s, 1H), 7.77 (d, *J* = 7.1 Hz, 2H), 7.70 (d, *J* = 7.1 Hz, 2H), 7.54 − 7.48 (m, 2H), 7.40 − 7.29 (m, 3H), 3.87 (s, 2H), 3.03 (s, 3H), 2.04 (s, 3H). Carbon-13 NMR (DMSO-*d_6_*) *δ* 172.0, 170.5, 168.6, 166.3, 162.1, 152.9, 137.1, 135.5, 131.6, 130.8, 129.6, 126.7, 124.0, 123.8, 120.3, 107.9, 89.5, 36.4, 34.5, 11.5. MS m/z (%): 551.39 (M^+^, 52.67). Anal. Calcd (551.42): C, 52.28; H, 3.47; Br, 14.49; N, 15.24; S, 5.82. Found: C, 52.40; H, 3.48; Br, 14.55; N, 15.14; S, 5.77%.

2-((5-Cyano-4–(4-methoxyphenyl)-6-oxo-1,6-dihydropyrimidin-2-yl)thio)-*N*-(1,5-dimethyl-3-oxo-2-phenyl-2,3-dihydro-1*H*-pyrazol-4-yl)acetamide (**8d**)

White coloured powder; (0.116 g, 77%). M.p. 166–168 °C. IR (νmax/cm^−1^): 3482, 3301 (NHs), 2219 (CN), 1687, 1655 (C = Os). Proton NMR (DMSO-*d_6_*) *δ* 9.55 (s, 1H), 8.08 (d, *J* = 8.5 Hz, 2H), 7.50 (d, *J* = 7.1 Hz, 2H), 7.41 − 7.30 (m, 3H), 7.10 (d, *J* = 8.5 Hz, 2H), 4.22 (s, 2H), 3.84 (s, 3H), 3.00 (s, 3H), 1.87 (s, 3H). Carbon-13 NMR (DMSO-*d_6_*) *δ* 171.9, 171.3, 169.1, 167.7, 162.1, 159.8, 153.5, 140.3, 129.7, 129.1, 129.2, 127.9, 124.3, 120.4, 114.2, 107.3, 89.2, 55.8, 36.2, 25.6, 11.2. MS m/z (%): 502.41 (M^+^, 34.41). Anal. Calcd (502.54): C, 59.75; H, 4.41; N, 16.72; S, 6.38. Found: C, 59.64; H, 4.48; N, 16.86; S, 6.22%.

### Anti-inflammatory studies

Twelve potential anti-inflammatory compounds derived from antipyrine were screened for their potential inhibitory activity against COX-1 and COX-2 enzymes and for their selectivity profiles using CLX as a reference drug. Then, the identified hit’' compounds with high COX-2 selectivity compared to CLX were tested *in vivo* for their anti-inflammatory efficacy against paw edema in rats induced by carrageenan and their potential underlying mechanisms were further investigated.

#### In vitro cyclooxygenase (COX) inhibition assay

This assay examined the inhibitory potency of the target structures towardboth COX isoforms via colorimetric screening assay kit (Cayman Chemical, MI, United States) based on the directions provided by the manufacturer. ELISA reader Thermo Scientific Multiskan® EX (Thermo scientific, United States) was used. IC_50_ (concentration at which there was 50% inhibition) values were calculated using GraphPad Prism 8 analysis software (Graph-Pad, San Diego, CA, United States) to form the dose–response curves of eight concentrations of each test compound. Serial dilutions of CLX, as a reference standard, and tested compounds at concentrations of 1000, 300, 100, 30, 10, 3, 1, and 0.30 nM were used in the assay. IC_50_ of the tested compounds were determined using three different experiments. The selectivity (SI) of each ompound was measured using the COX-2/COX-1 ratio, which compares the IC_50_ of COX-2 and COX-1.

#### In vivo evaluation of the anti-inflammatory activity

The anti-inflammatory activity of the two selected compounds from the above screening, **4b** and **4c** was scrutinized *in vivo* using a paw edema model produced by carrageenan. Male Sprague Dawley rats (180–200 g) were obtained from Vacsera (Giza, Egypt) and maintained at rtunfettered access to food and water in a light-controlled space with a twelve-hour light/dark cycle. The research ethics committee at Mansoura University, Egypt examined and authorized the experiments (code number: 2022–78).

##### Experimental design

After overnight fasting, five groups of rats were randomly divided (*n* = 5). Group I (Control): injected only with the DMSO solution (1.2 ml/kg, *i.p*, 10% v/v of DMSO in sterile 0.9% saline solution). Group II (Carrageenan): injected with carrageenan [0.1 ml of a freshly prepared solution of carrageenan (1% w/v in sterile 0.9% saline solution) into the sub-plantar region of the right hind paw] and the DSMO solution. Group III (CLX): injected the standard drug CLX (30 mg/kg*, i.p*, 2.5% w/v in the DMSO solution). Group IV (**4 b**) injected the test drug **4 b** (36 mg/kg, *i.p*, 2% w/v in the vehicle). Group V (**4c**): injected the test drug **4c** (40 mg/kg, *i.p*, 2% w/v in the DMSO solution). The selected doses of **4 b** and **4c** were equimolar to 30 mg/kg CLX. One hour after treatments, edema caused by carrageenan injection as described above. A digital vernier caliper (Elora, Germany) was used to measure the thickness of the paw right prior to injection of carrageenan and thereafter at 1, 2, 3, 4, and 5 h.

#### Determination of paw edoema and edoema inhibition rate

The paw edoema (E%) and edoema inhibition rate (I%) were calculated as follow:

$$E\%=\frac{B-A}{A}\text{×}100,$$

*A:* paw thickness (mm) just before carrageenan injection and *B:* paw thickness (mm) at different time intervals after carrageenan injection:

$$I\%=\frac{E_{c}-E_{t}}{E_{c}}\times 100,$$

*E*_c_: mean paw edoema of the carrageenan group and *E*_t_ is the paw odema of the tested groups.

##### Paw homogenate preparation

After five hours from carrageenan injection, the rats were euthanized using surgical dislocation with removal of the inflamed paws. Tissues of rat hind-paw’s pad were collected, and 5-mm pieces were homogenised in iced phosphate buffer saline (pH 7.4) on ice. Next, the prepared homogenate was centrifuged (10,000 g, 15 min, 4 °C) and the obtained supernatant was collected for additional analysis. The remaining tissue was fixed in formalin (10% v/v) for 24 h after which it was inserted in paraffin wax.

#### Histopathological and immunohistochemical evaluation

Paraffinized tissue sections were dewaxed in xylene and mounted on slides then discoloured with hematoxylin-eosin (H&E) for histopathological evaluation of inflammation. Inflammation score from 0 to 5 was given as follows: 0,1,2,3,4, and 5 if there is no, mild, mild/moderate, moderate, moderate/severe, and severe inflammation, respectively.^48^ The other set of sections were immunostained to examine expression levels of COX-2 and nuclear factor kappa B (NF-κB p65) using monoclonal antibodies (Thermo Fisher Scientific, MA, United States) and as per the guidelines provided by the manufacturer. Immunostained sections were scored as follows: 0,1, 2 and 3 if there is no, weak, moderate, and strong staining effect, respectively.^49^

#### Determination of PGE2 and TNF-α tissue levels

Levels of prostaglandin E2 (PGE2) and tumour necrosis factor-alpha (TNF-α) were measured in paw tissue homogenate using ELISA kits (Cusabio Technology LLC, TX, United States) and as per the guidelines provided by the manufacturer.

#### Oxidative and nitrosative biomarkers measurements

Contents of malondialdehyde (MDA) as a lipid peroxidation product, and nitric oxide (NO) as anitrosative product, were measured in paw tissue homogenate using colorimetric commercial kits (Biodiagnostic, Giza, Egypt) and as per the guidelines provided by the manufacturer.

### Statistical analysis

Using GraphPad Prism 8 analysis software (Graph-Pad, San Diego, CA, USA), data were expressed as mean ± S.E (*n* = 5) and analysed *via* ANOVA test followed by Tukey–Kramer multiple comparison test as *post hoc* test. Kruskal–Wallis test by rank followed by Dunn’s multiple comparison test was applied for scores of histopathological and immunohistochemistry.

### Computational details

Molecular docking studies were performed in Maestro suite release 2020–3 employing Glide software.^50^^,^^51^ The 3D structures of *h*COX-1 and *h*COX-2 (PDB IDs 6Y3C and 5KIR, respectively) were retrieved from the Protein Data Bank and acquiesced to the Protein Preparation Wizard protocol executed in Maestro for obtaining appropriate structures for the computational studies. The structures of the ligands were built in Maestro employing the available drawing tools, while energy minimization was achieved by using MacroModel program with OPLS-2005 as the force field. Subsequently, LigPrep application (LigPrep, Schrödinger, LLC, New York, NY, release 20120–3) was used for generating the most probable ionization state of the molecules at cellular pH. Molecular docking experiments were performed employing the scoring function extra precision (XP). Energy grids using a protein atom scaling factor of 1.0 Å were obtained using default values. The cubic box used for docking calculation, for each protein, was prepared starting from the middle of thepocket. Fifty poses were considered for the post-docking minimization procedure. The number of poses entered to post-docking minimization was set to 50. The validation of the docking protocol is described in the Supplementary data file.

## Supplementary Material

Supplemental Material
