# Supplementary material for: Design, synthesis, and biological investigation of oxadiazolyl, thiadiazolyl, and pyrimidinyl linked antipyrine derivatives as potential non-acidic anti-inflammatory agents
Source: J Enzyme Inhib Med Chem. 2023 Jan 12;38(1):2162511. doi: 10.1080/14756366.2022.2162511 (PMC9848286; doi:10.1080/14756366.2022.2162511)
Supplement: Supplemental Material [file IENZ_A_2162511_SM8136.pdf]

Design, Synthesis and Biological Investigation of Oxadiazolyl, Thiadiazolyl and Pyrimidinyl Linked Antipyrine Derivatives as Potential Non-Acidic Anti-Inflammatory Agents

Mohammad M. Al-Sanea[1,†,*], Abdelrahman Hamdi[2,†], Simone Brogi[3], Samar S. Tawfik[2], Dina I. A. Othman[2], Mahmoud Elshal[4], Hidayat Ur Rahman[5], Della G. T. Parambi[1], Rehab M. Elbargisy[6], Samy Selim[7], Ehab M. Mostafa[8], Ahmed A. B. Mohamed[9, †,*]

[1] Department of Pharmaceutical Chemistry, College of Pharmacy, Jouf University, Sakaka 72341, Saudi Arabia

[2] Department of Pharmaceutical Organic Chemistry, Faculty of Pharmacy, Mansoura University, Mansoura 35516, Egypt

[3] Department of Pharmacy, University of Pisa, Via Bonanno 6, 56126 Pisa, Italy

[4] Department of Pharmacology and Toxicology, Faculty of Pharmacy, Mansoura University, Mansoura 35516, Egypt.

[5] Department of Clinical Pharmacy, College of Pharmacy, Jouf University, Sakaka, Aljouf-72341, Saudi Arabia

[6] Department of Pharmaceutics, College of Pharmacy, Jouf University, Sakaka, Al-Jouf, Saudi Arabia

[7] Department of Clinical Laboratory Sciences, College of Applied Medical Sciences, Jouf University, Sakaka 72341, Saudi Arabia: sabdulsalam@ju.edu.sa

[8] Department of Pharmacognosy, College of Pharmacy, Jouf University, Sakaka 72341, Saudi Arabia

[9] Department of Medicinal Chemistry, Faculty of Pharmacy, Mansoura University, Mansoura 35516, Egypt

[*] Corresponding authors at: Department of Pharmaceutical Chemistry, College of Pharmacy, Jouf University, Sakaka 72341, Saudi Arabia: mmalsanea@ju.edu.sa; Department of Medicinal Chemistry, Faculty of Pharmacy, Mansoura University, Mansoura 35516, Egypt: ahmed_smt@yahoo.com

[†] Contributed equally.

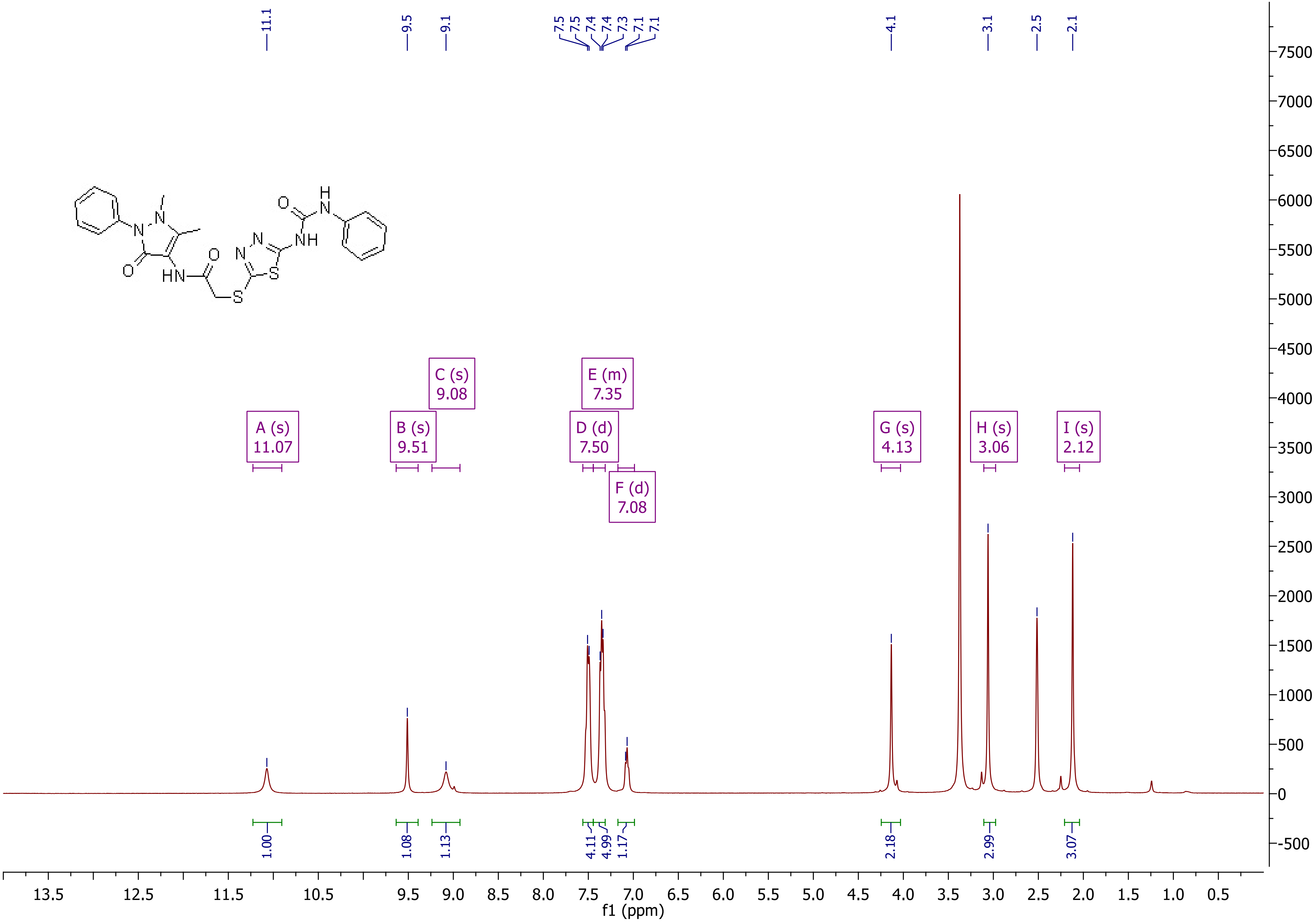

11.1
9.5
9.1
7.5
7.5
7.4
7.4
7.3
7.1
7.1
4.1
3.1
2.5
2.1
A (s)
11.07
B (s)
9.51
C (s)
9.08
D (d)
7.50
E (m)
7.35
F (d)
7.08
G (s)
4.13
H (s)
3.06
I (s)
2.12
1.00
1.08
1.13
4.11
4.99
1.17
2.18
2.99
3.07
f1 (ppm)
13.5
12.5
11.5
10.5
9.5
9.0
8.5
8.0
7.5
7.0
6.5
6.0
5.5
5.0
4.5
4.0
3.5
3.0
2.5
2.0
1.5
1.0
0.5
7500
7000
6500
6000
5500
5000
4500
4000
3500
3000
2500
2000
1500
1000
500
0
-500

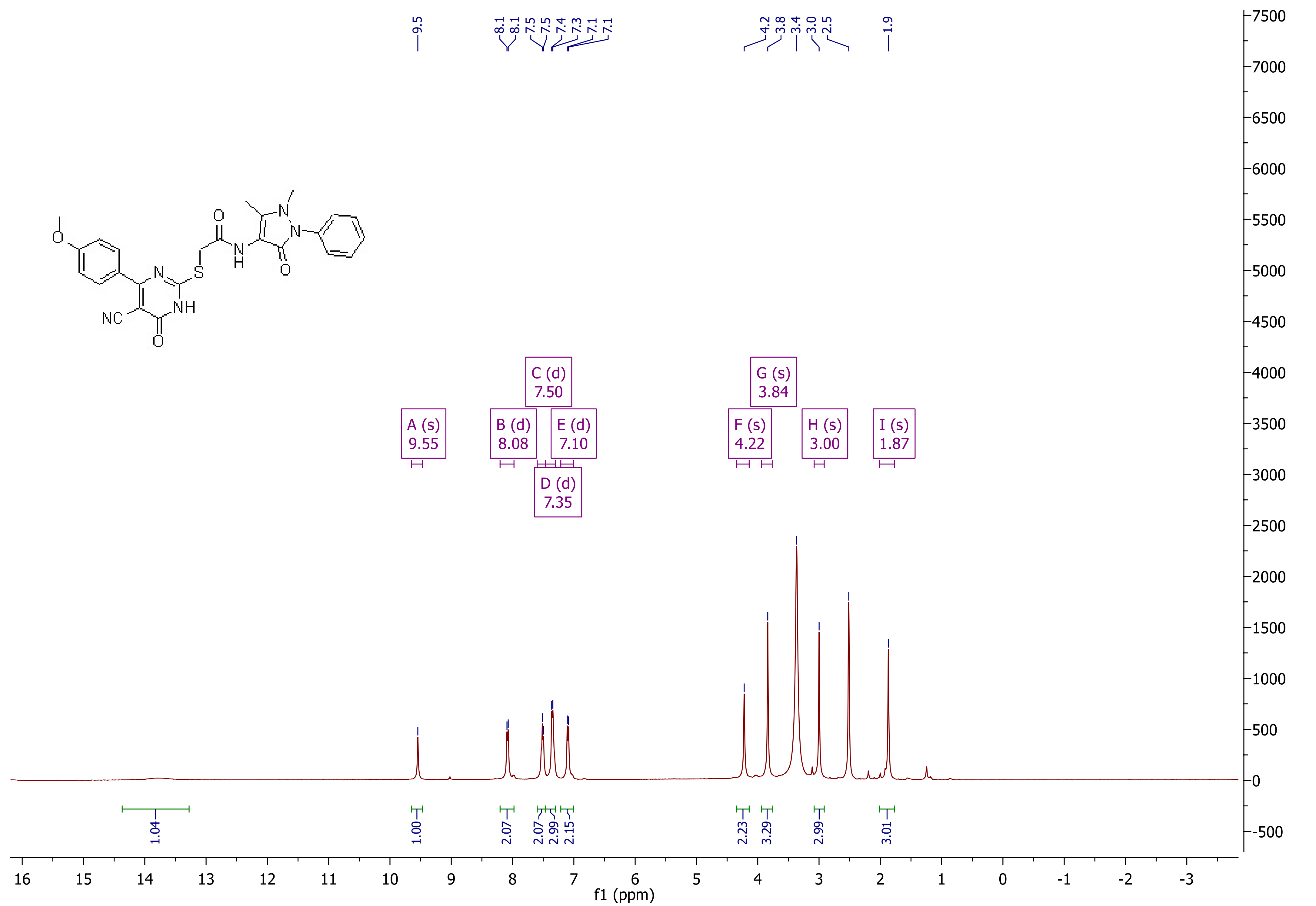

9.5
8.1
8.1
7.5
7.5
7.4
7.3
7.1
7.1
4.2
3.8
3.4
3.0
2.5
1.9
NC
NH
N
S
O
H
N
A (s) 9.55
B (d) 8.08
C (d) 7.50
D (d) 7.35
E (d) 7.10
F (s) 4.22
G (s) 3.84
H (s) 3.00
I (s) 1.87
1.04
1.00
2.07
2.07
2.99
2.15
2.23
3.29
2.99
3.01
16 15 14 13 12 11 10 9 8 7 6 5 4 3 2 1 0 -1 -2 -3
f1 (ppm)
7500 7000 6500 6000 5500 5000 4500 4000 3500 3000 2500 2000 1500 1000 500 0 -500

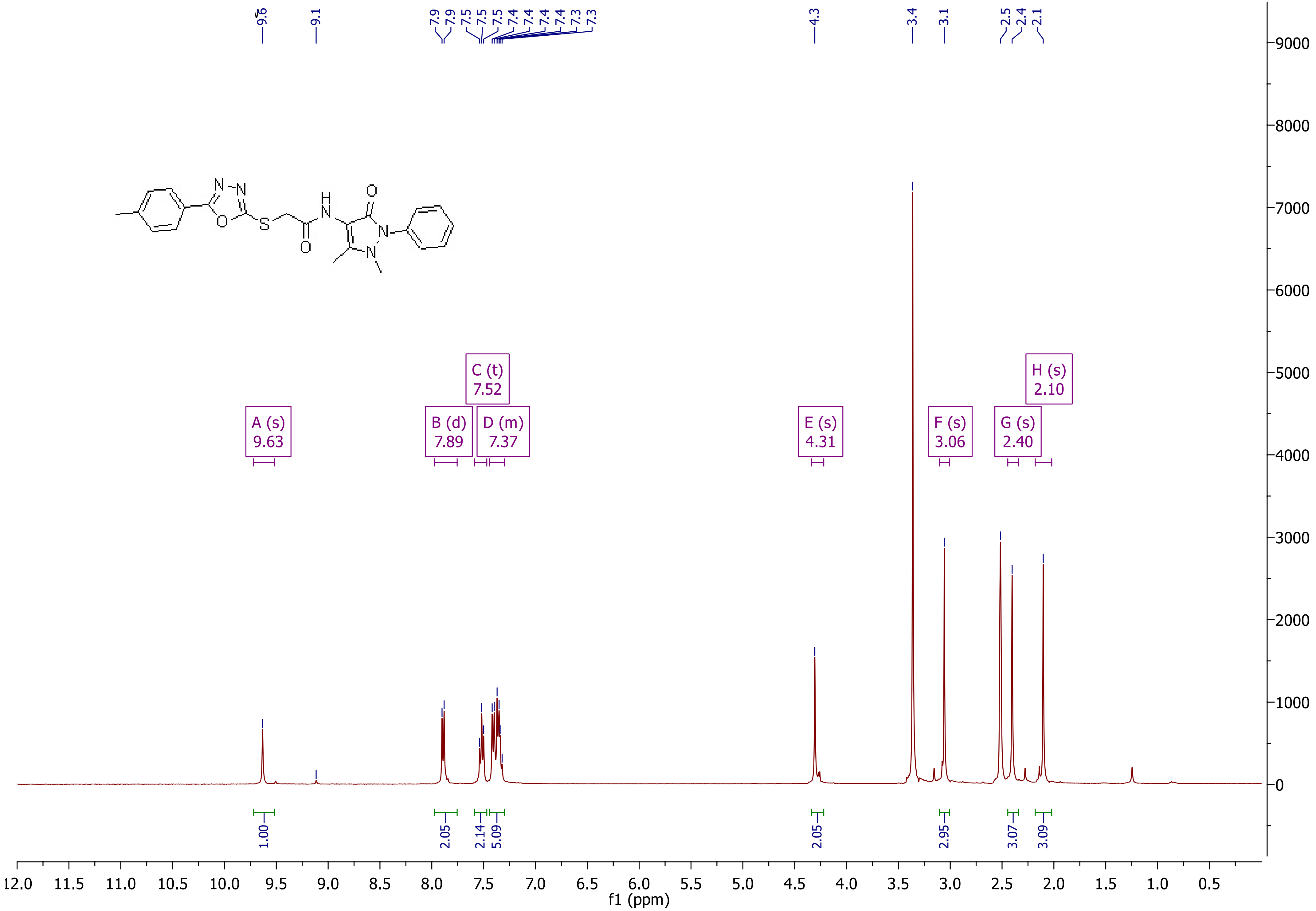
9.6
9.1
7.9
7.9
7.5
7.5
7.5
7.4
7.4
7.4
7.4
7.3
7.3
4.3
3.4
3.1
2.5
2.4
2.1
A (s)
9.63
B (d)
7.89
C (t)
7.52
D (m)
7.37
E (s)
4.31
F (s)
3.06
G (s)
2.40
H (s)
2.10
1.00
2.05
2.14
5.09
2.05
2.95
3.07
3.09
9000
8000
7000
6000
5000
4000
3000
2000
1000
0
12.0
11.5
11.0
10.5
10.0
9.5
9.0
8.5
8.0
7.5
7.0
6.5
6.0
5.5
5.0
4.5
4.0
3.5
3.0
2.5
2.0
1.5
1.0
0.5
f1 (ppm)

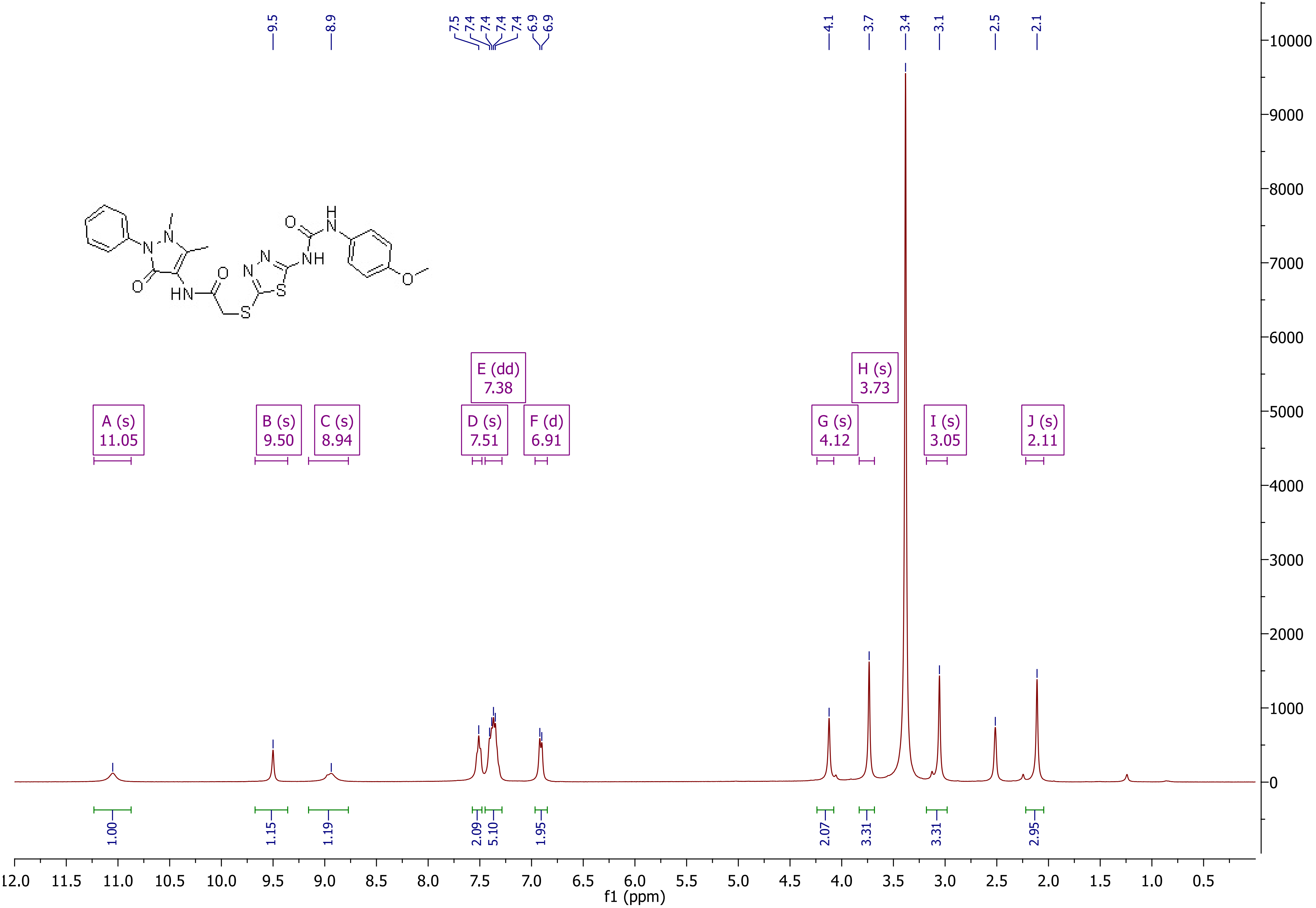

9.5
8.9
7.5
7.4
7.4
7.4
7.4
6.9
6.9
4.1
3.7
3.4
3.1
2.5
2.1
A (s)
11.05
B (s)
9.50
C (s)
8.94
D (s)
7.51
E (dd)
7.38
F (d)
6.91
G (s)
4.12
H (s)
3.73
I (s)
3.05
J (s)
2.11
1.00
1.15
1.19
2.09
5.10
1.95
2.07
3.31
3.31
2.95
12.0
11.5
11.0
10.5
10.0
9.5
9.0
8.5
8.0
7.5
7.0
6.5
6.0
5.5
5.0
4.5
4.0
3.5
3.0
2.5
2.0
1.5
1.0
0.5
f1 (ppm)
10000
9000
8000
7000
6000
5000
4000
3000
2000
1000
0

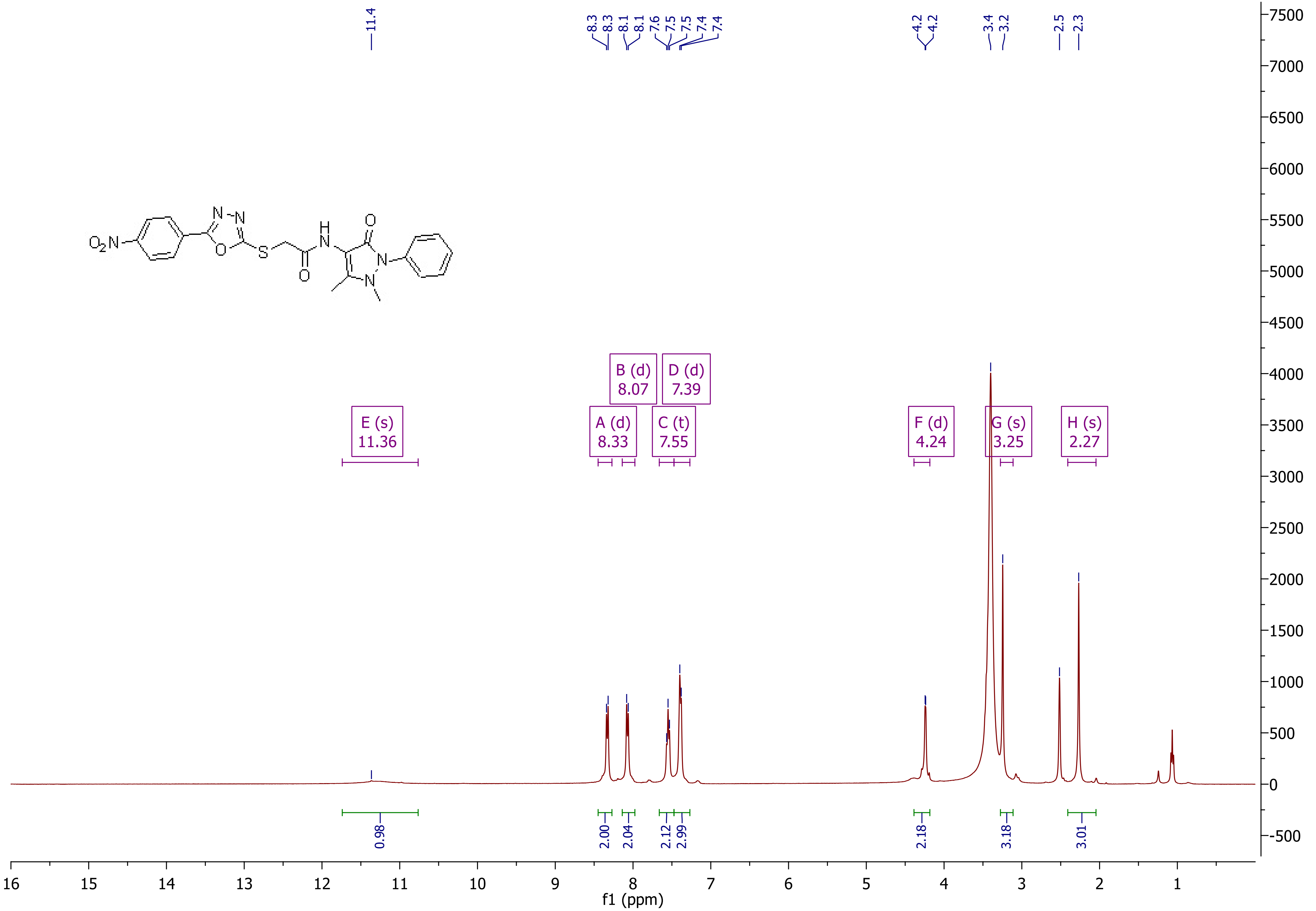
11.4
8.3
8.3
8.1
8.1
7.6
7.5
7.5
7.4
7.4
4.2
4.2
3.4
3.2
2.5
2.3
B (d) 8.07
D (d) 7.39
E (s) 11.36
A (d) 8.33
C (t) 7.55
F (d) 4.24
G (s) 3.25
H (s) 2.27
0.98
2.00
2.04
2.12
2.99
2.18
3.18
3.01
f1 (ppm)

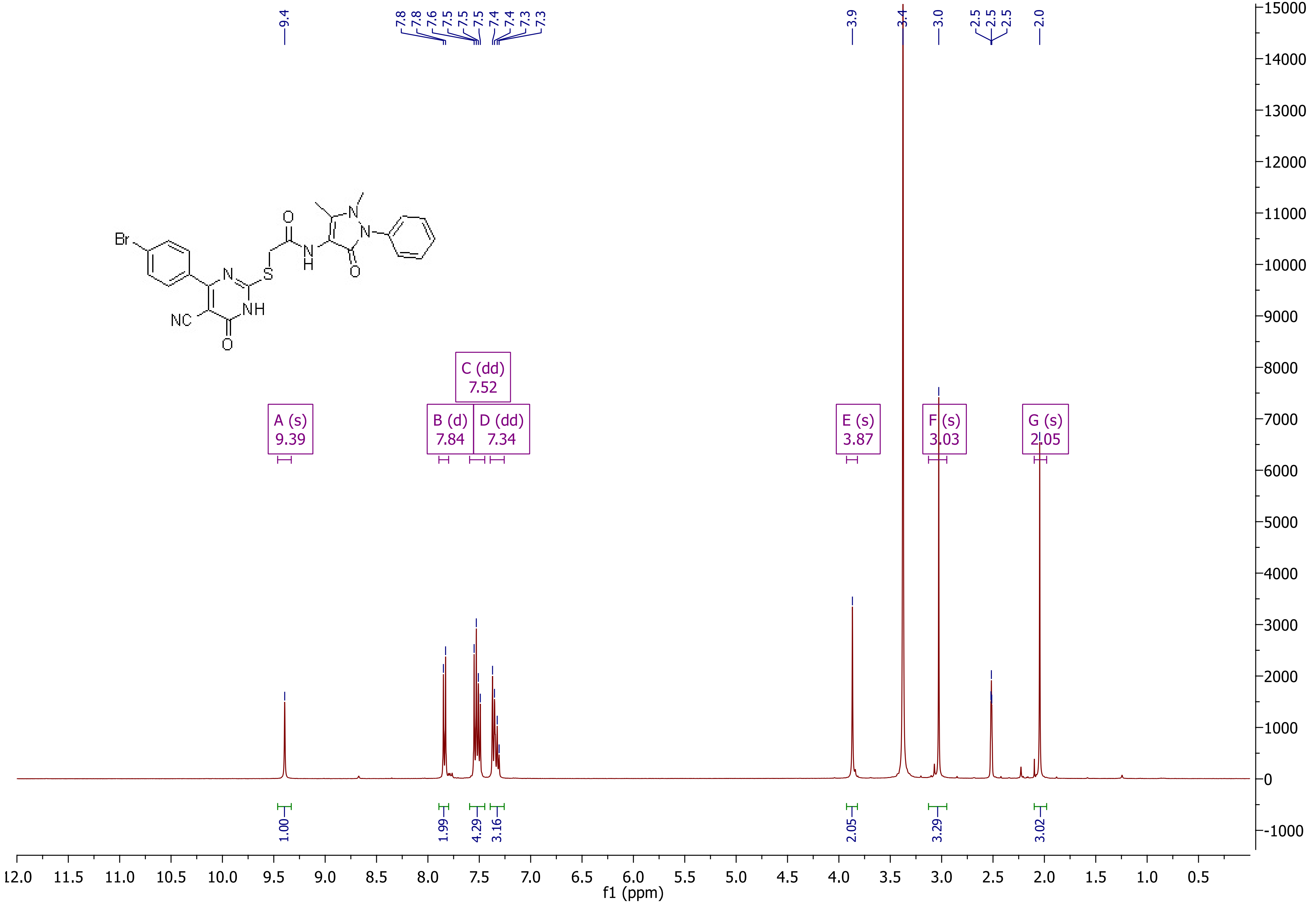

A (s)
9.39
B (d)
7.84
C (dd)
7.52
D (dd)
7.34
E (s)
3.87
F (s)
3.03
G (s)
2.05
1.00
1.99
4.29
3.16
2.05
3.29
3.02
f1 (ppm)
Br
NC
NH
N
S
O
H

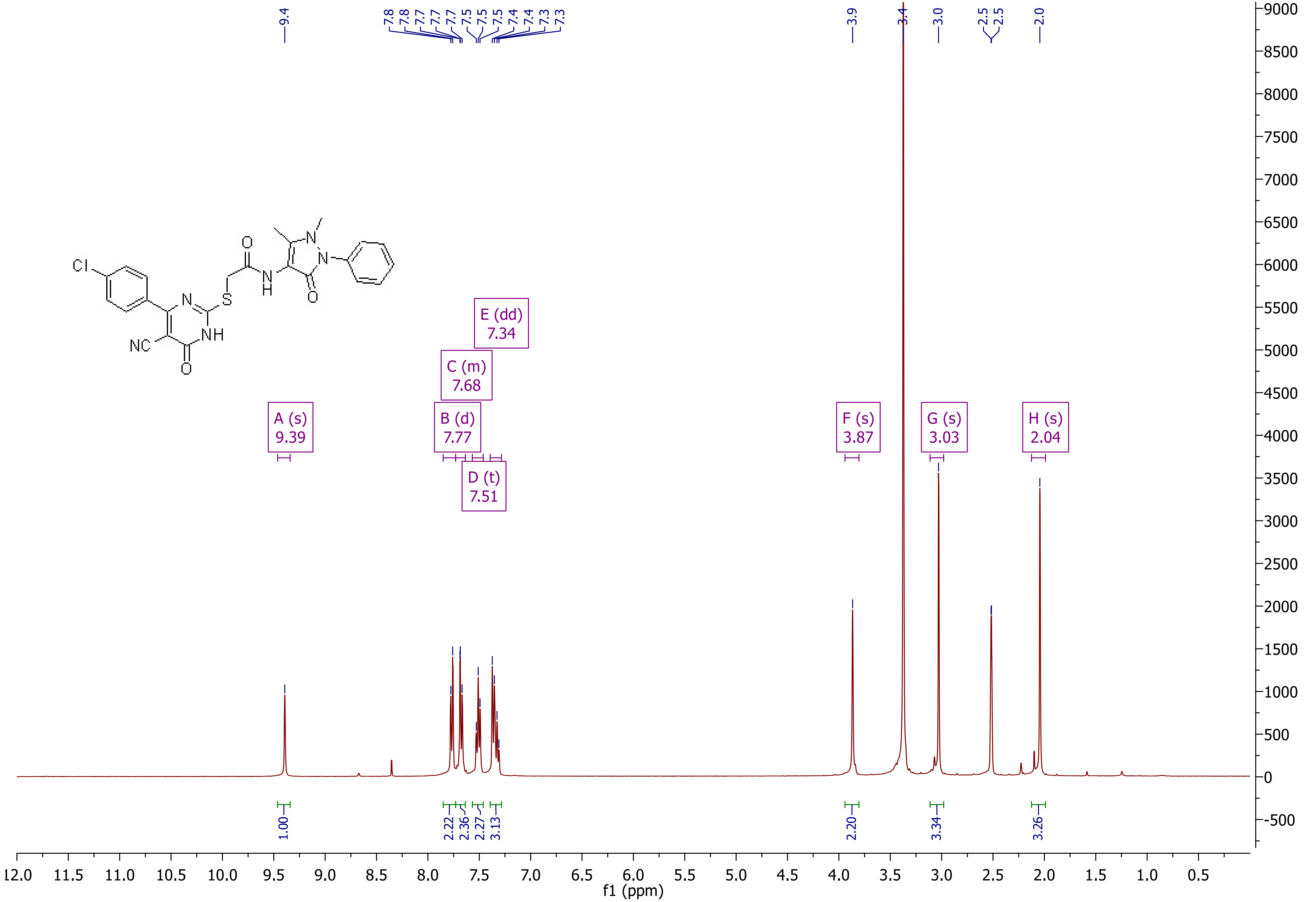

9.4
7.8
7.8
7.7
7.7
7.7
7.5
7.5
7.5
7.4
7.4
7.3
7.3
3.9
3.4
3.0
2.5
2.5
2.0
A (s)
9.39
B (d)
7.77
C (m)
7.68
D (t)
7.51
E (dd)
7.34
F (s)
3.87
G (s)
3.03
H (s)
2.04
1.00
2.22
2.36
2.27
3.13
2.20
3.34
3.26
Cl
N
S
NH
NC
O
O
N
H
N
N
O
f1 (ppm)

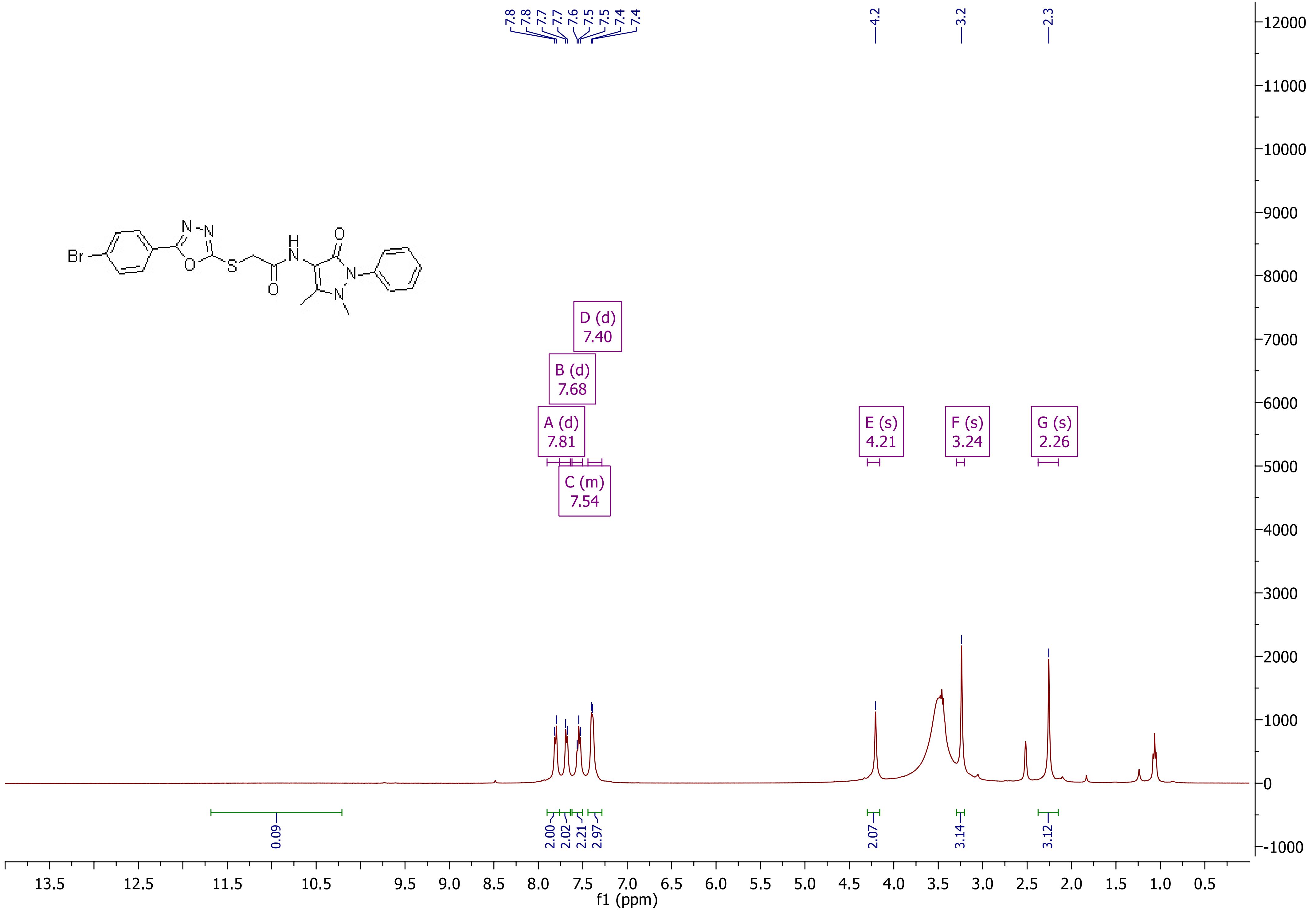

7.8
7.8
7.7
7.7
7.6
7.5
7.5
7.4
7.4
4.2
3.2
2.3
Br
N
N
O
S
H
N
O
O
N
N
D (d)
7.40
B (d)
7.68
A (d)
7.81
C (m)
7.54
E (s)
4.21
F (s)
3.24
G (s)
2.26
0.09
2.00
2.02
2.21
2.97
2.07
3.14
3.12
12000
11000
10000
9000
8000
7000
6000
5000
4000
3000
2000
1000
0
-1000
13.5
12.5
11.5
10.5
9.5
9.0
8.5
8.0
7.5
7.0
6.5
6.0
5.5
5.0
4.5
4.0
3.5
3.0
2.5
2.0
1.5
1.0
0.5
f1 (ppm)

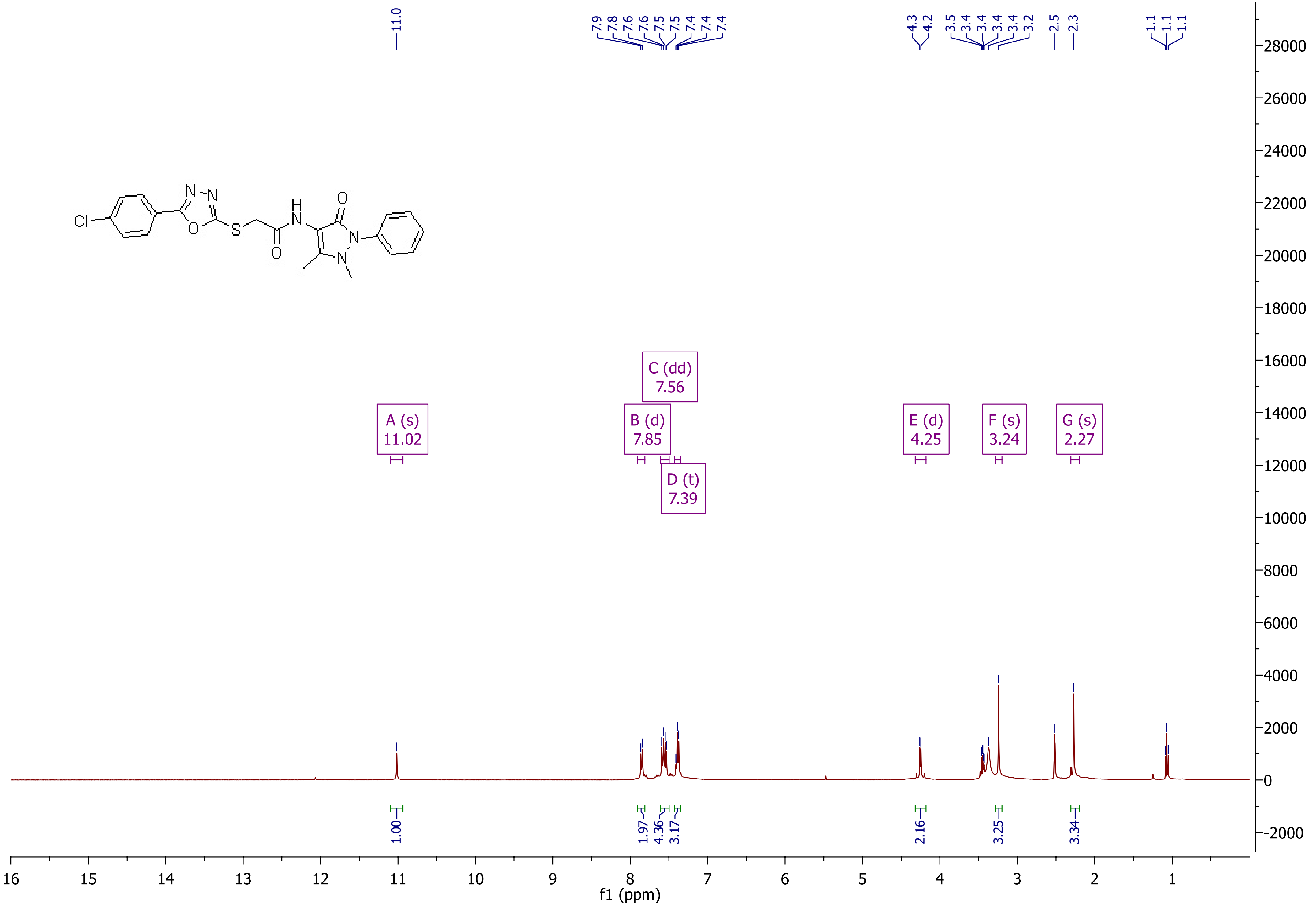

11.0
7.9 7.8 7.6 7.6 7.5 7.5 7.4 7.4 7.4
4.3 4.2
3.5 3.4 3.4 3.4 3.4 3.2
2.5
2.3
1.1 1.1 1.1
A (s) 11.02
B (d) 7.85
C (dd) 7.56
D (t) 7.39
E (d) 4.25
F (s) 3.24
G (s) 2.27
1.00
1.97
4.36
3.17
2.16
3.25
3.34
f1 (ppm)

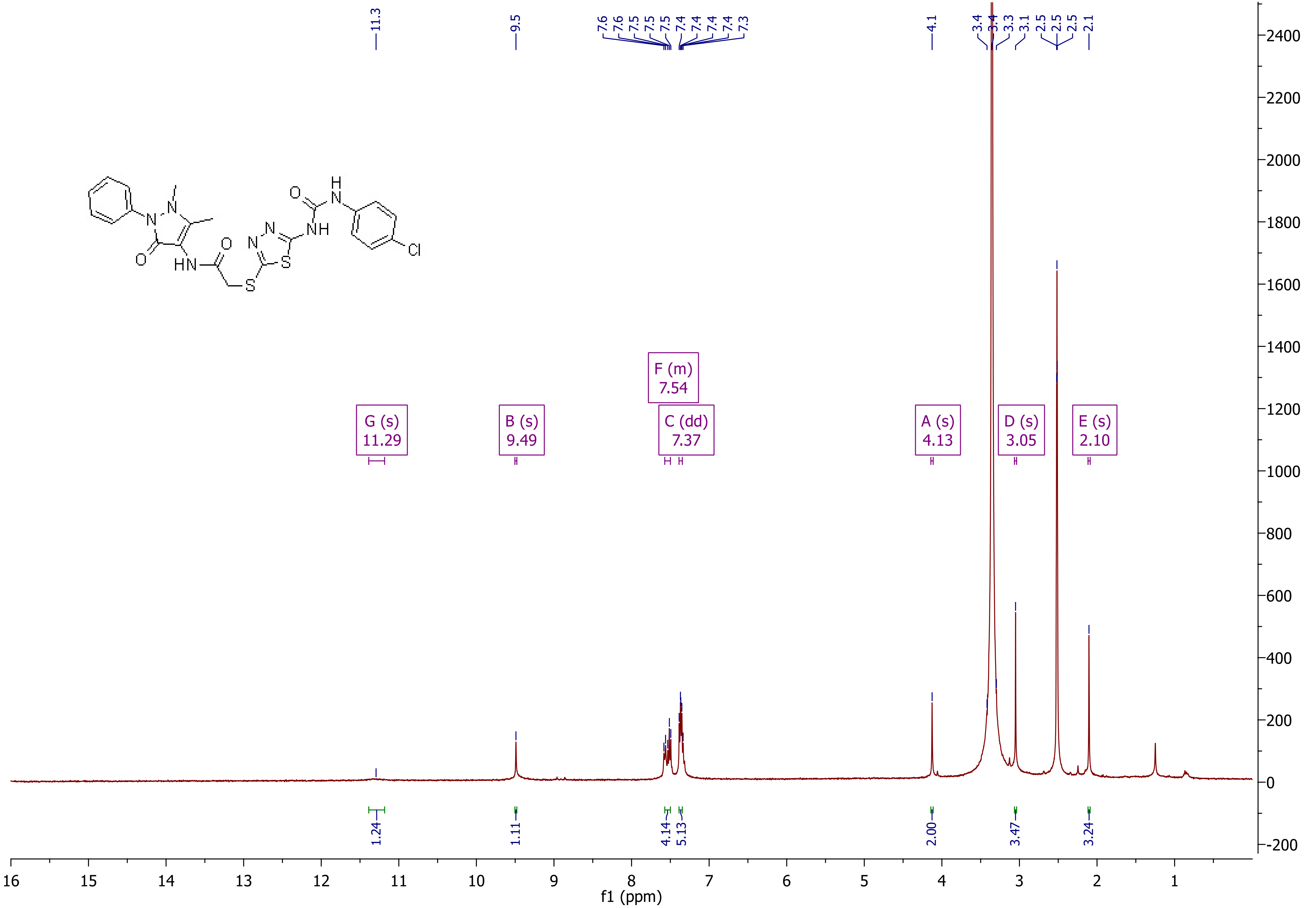

11.3
9.5
7.6
7.6
7.5
7.5
7.5
7.4
7.4
7.4
7.4
7.3
4.1
3.4
3.4
3.3
3.1
2.5
2.5
2.5
2.1
F (m)
7.54
G (s)
11.29
B (s)
9.49
C (dd)
7.37
A (s)
4.13
D (s)
3.05
E (s)
2.10
1.24
1.11
4.14
5.13
2.00
3.47
3.24
O
N
N
HN
O
S
N
N
S
NH
O
H
N
Cl
16
15
14
13
12
11
10
9
8
7
6
5
4
3
2
1
f1 (ppm)
2400
2200
2000
1800
1600
1400
1200
1000
800
600
400
200
0
-200

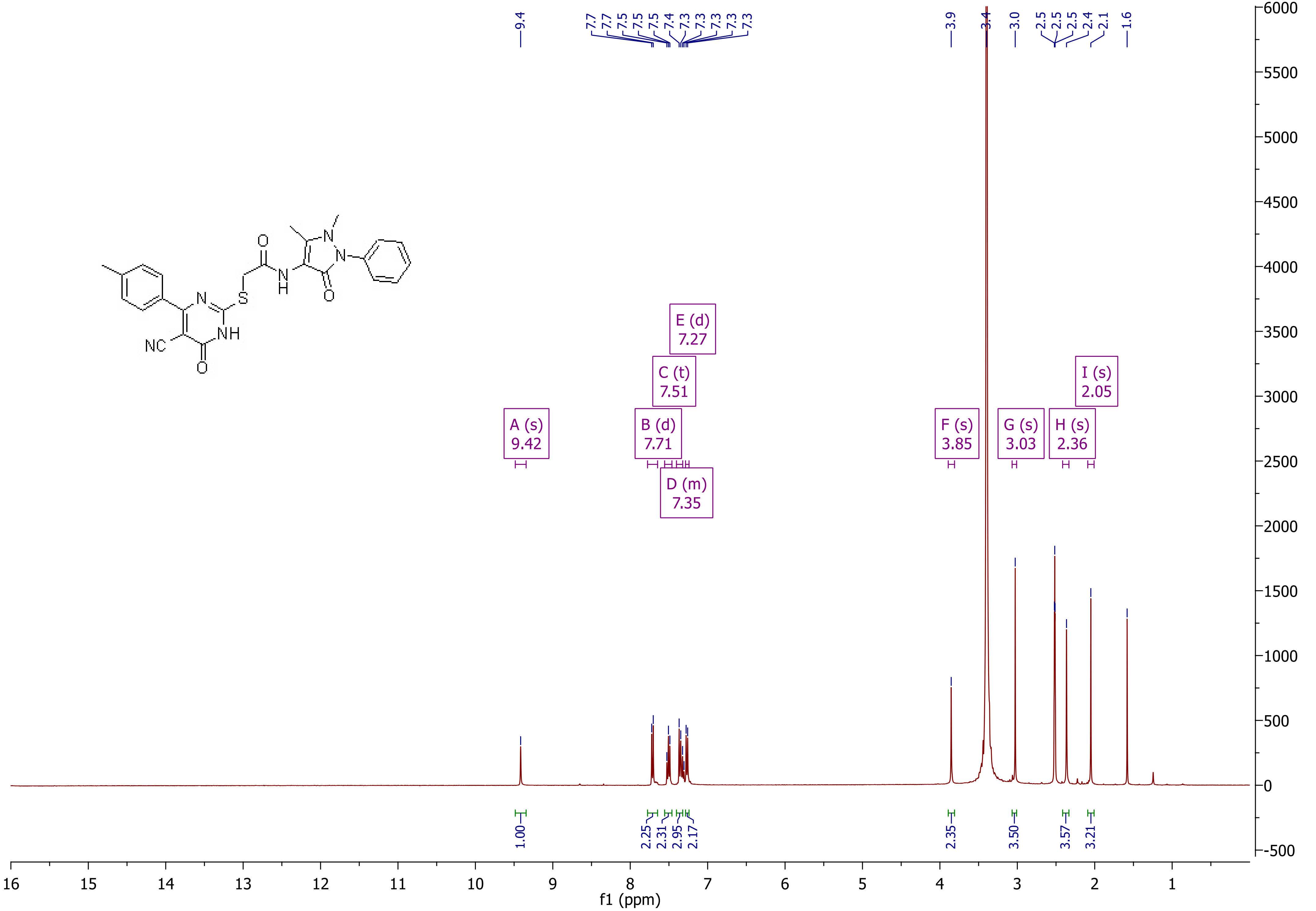

9.4
7.7
7.7
7.5
7.5
7.5
7.4
7.3
7.3
7.3
7.3
7.3
3.9
3.4
3.0
2.5
2.5
2.5
2.4
2.1
1.6
A (s)
9.42
B (d)
7.71
C (t)
7.51
D (m)
7.35
E (d)
7.27
F (s)
3.85
G (s)
3.03
H (s)
2.36
I (s)
2.05
1.00
2.25
2.31
2.95
2.17
2.35
3.50
3.57
3.21
NC
NH
N
S
O
H
f1 (ppm)
16 15 14 13 12 11 10 9 8 7 6 5 4 3 2 1
6000 5500 5000 4500 4000 3500 3000 2500 2000 1500 1000 500 0 -500

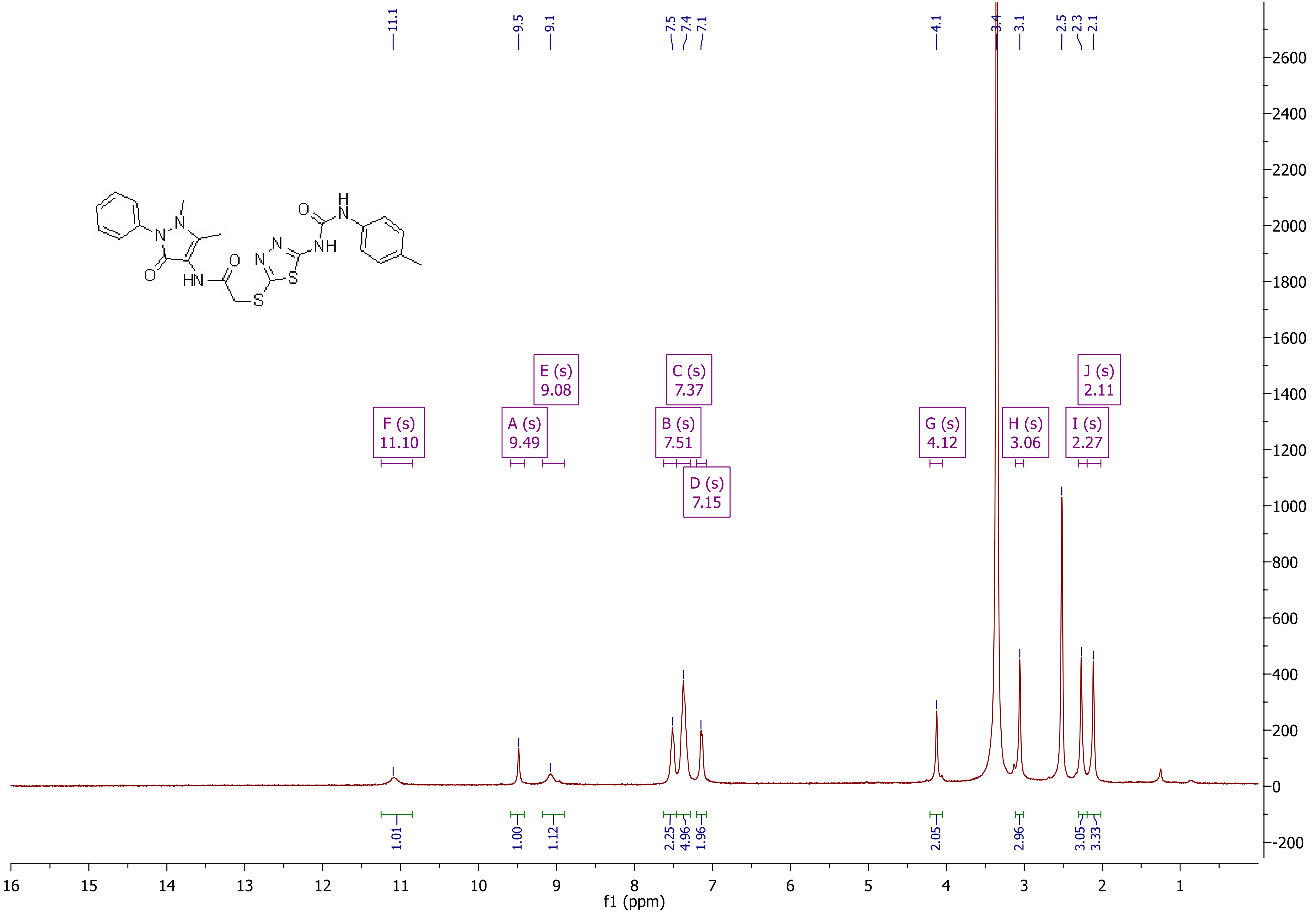
11.1
9.5
9.1
7.5
7.4
7.1
4.1
3.4
3.1
2.5
2.3
2.1
F (s)
11.10
A (s)
9.49
E (s)
9.08
B (s)
7.51
C (s)
7.37
D (s)
7.15
G (s)
4.12
H (s)
3.06
I (s)
2.27
J (s)
2.11
1.01
1.00
1.12
2.25
4.96
1.96
2.05
2.96
3.05
3.33
f1 (ppm)

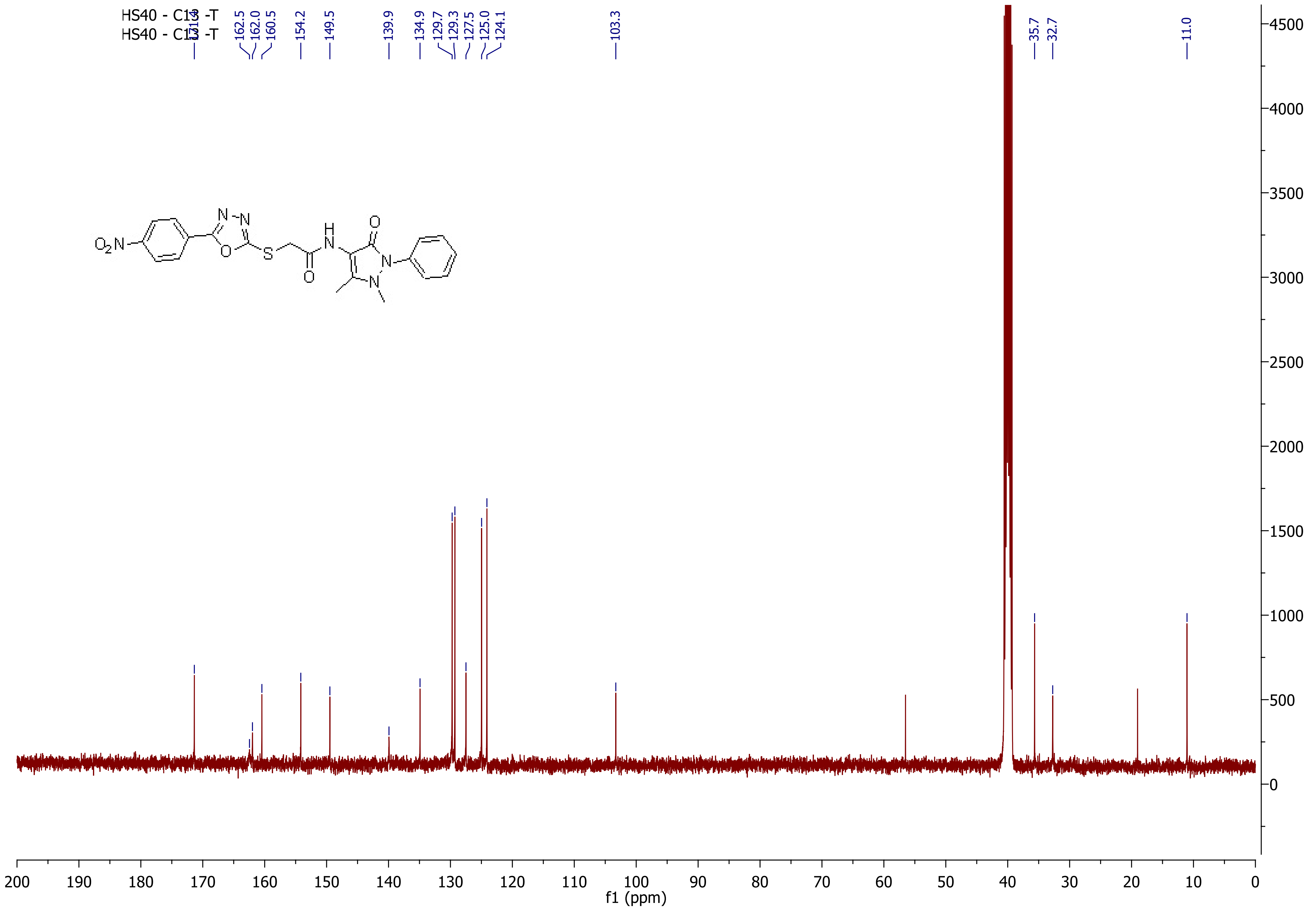
HS40 - C13 -T
HS40 - C13 -T
171.4
162.5
162.0
160.5
154.2
149.5
139.9
134.9
129.7
129.3
127.5
125.0
124.1
103.3
35.7
32.7
11.0
O_2N
N
N
O
S
H
N
O
O
N
N
f1 (ppm)

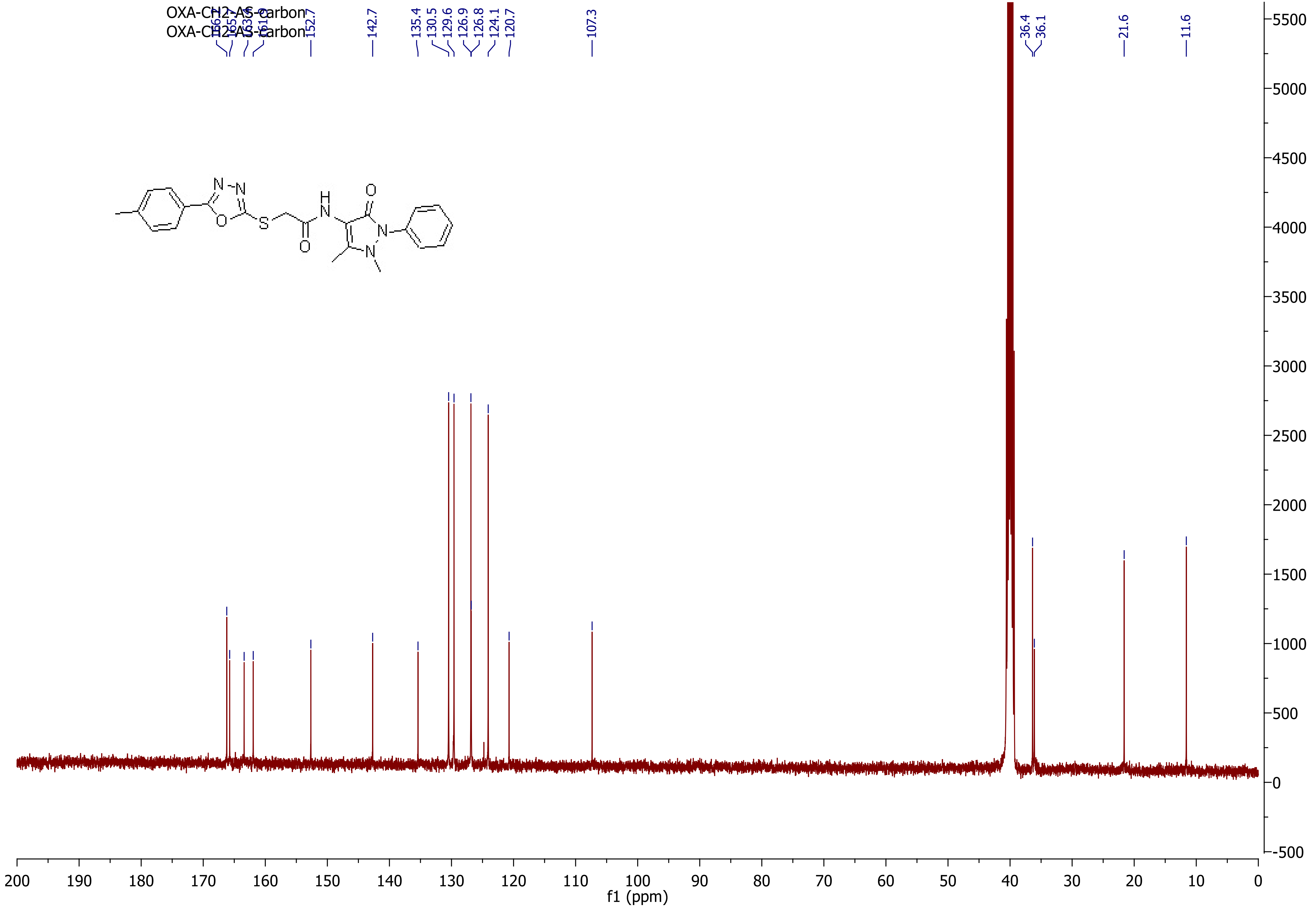
OXA-CH2-AS-carbon
OXA-CH2-AS-carbon
166.2
165.7
163.4
161.9
152.7
142.7
135.4
130.5
129.6
126.9
126.8
124.1
120.7
107.3
36.4
36.1
21.6
11.6
N
N
O
S
H
N
O
O
N
N
5500
5000
4500
4000
3500
3000
2500
2000
1500
1000
500
0
-500
200
190
180
170
160
150
140
130
120
110
100
90
80
70
60
50
40
30
20
10
0
f1 (ppm)

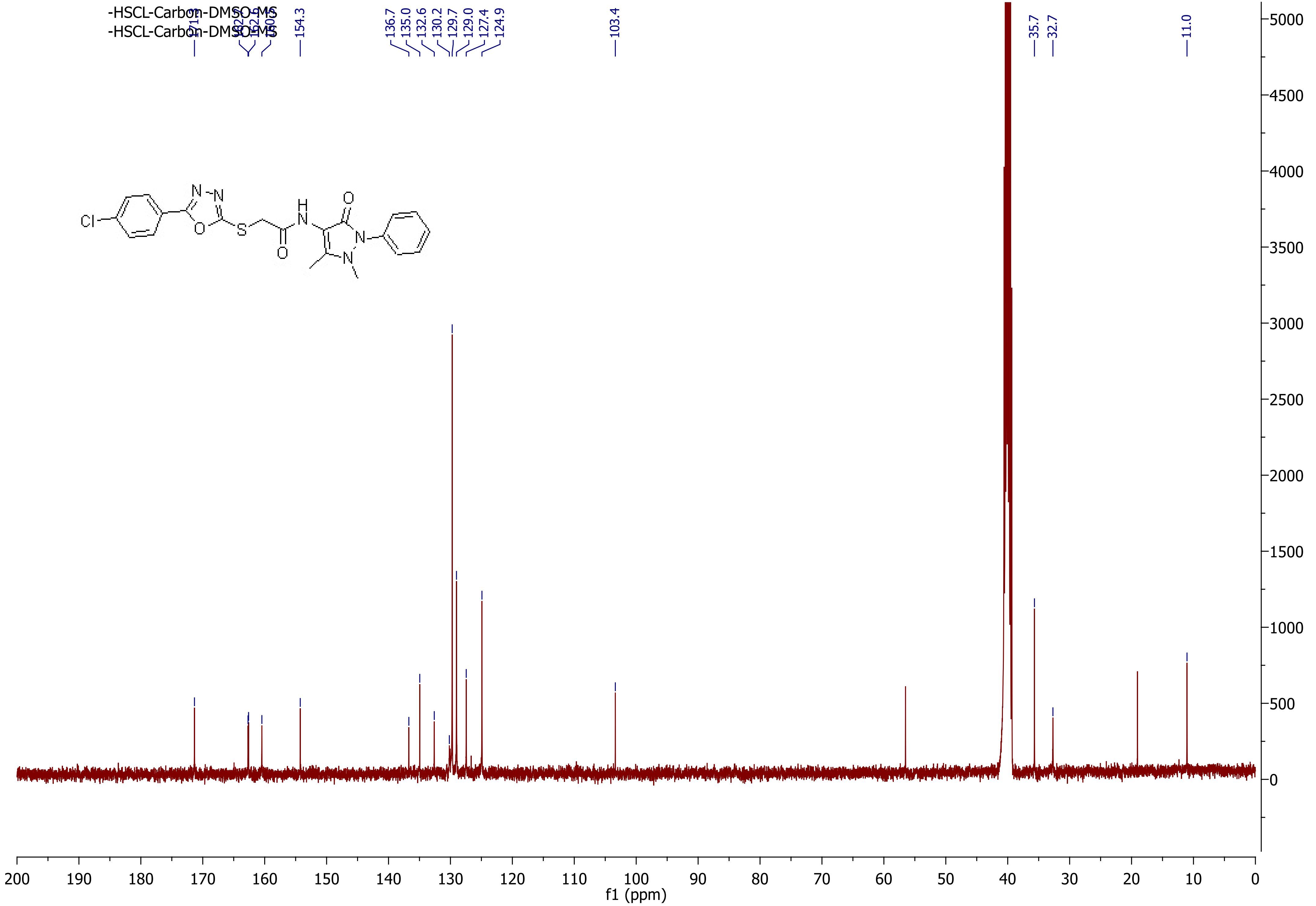
-HSCL-Carbon-DMSO-MS
-HSCL-Carbon-DMSO-MS
171.3
162.7
162.6
160.5
154.3
136.7
135.0
132.6
130.2
129.7
129.0
127.4
124.9
103.4
35.7
32.7
11.0
Cl
N
N
O
S
H
N
O
O
N
N
5000
4500
4000
3500
3000
2500
2000
1500
1000
500
0
200
190
180
170
160
150
140
130
120
110
100
90
80
70
60
50
40
30
20
10
0
f1 (ppm)

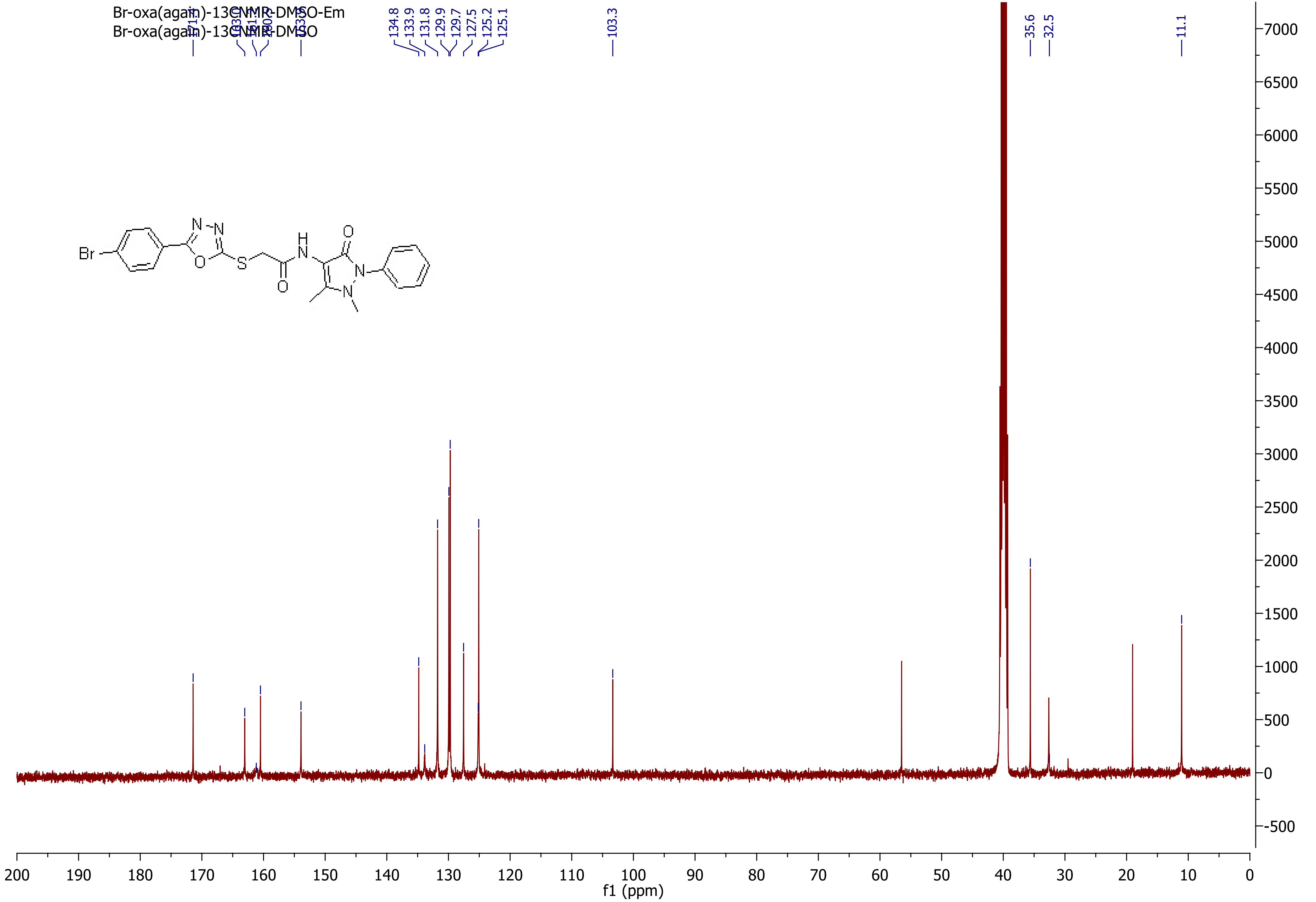
Br-oxa(again)-13CNMR-DMSO-Em
Br-oxa(again)-13CNMR-DMSO
134.8
133.9
131.8
129.9
129.7
127.5
125.2
125.1
103.3
35.6
32.5
11.1
Br
N
N
O
S
H
N
O
O
N
N
f1 (ppm)

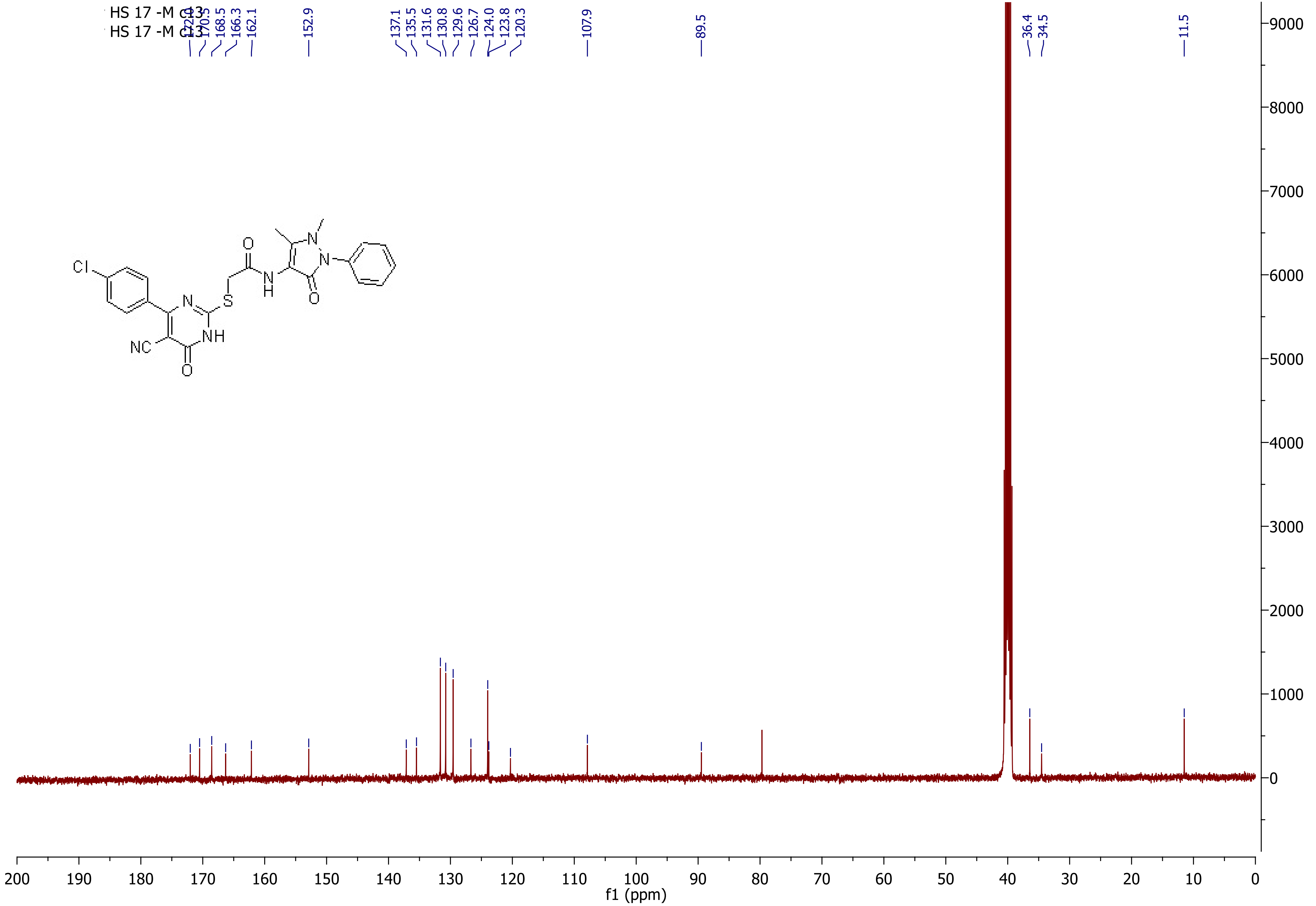

HS 17 -M c13
170.5
168.5
166.3
162.1
152.9
137.1
135.5
131.6
130.8
129.6
126.7
124.0
123.8
120.3
107.9
89.5
36.4
34.5
11.5
Cl
N
NH
S
O
NC
N
H
N
N
9000
8000
7000
6000
5000
4000
3000
2000
1000
0
200 190 180 170 160 150 140 130 120 110 100 90 80 70 60 50 40 30 20 10 0
f1 (ppm)

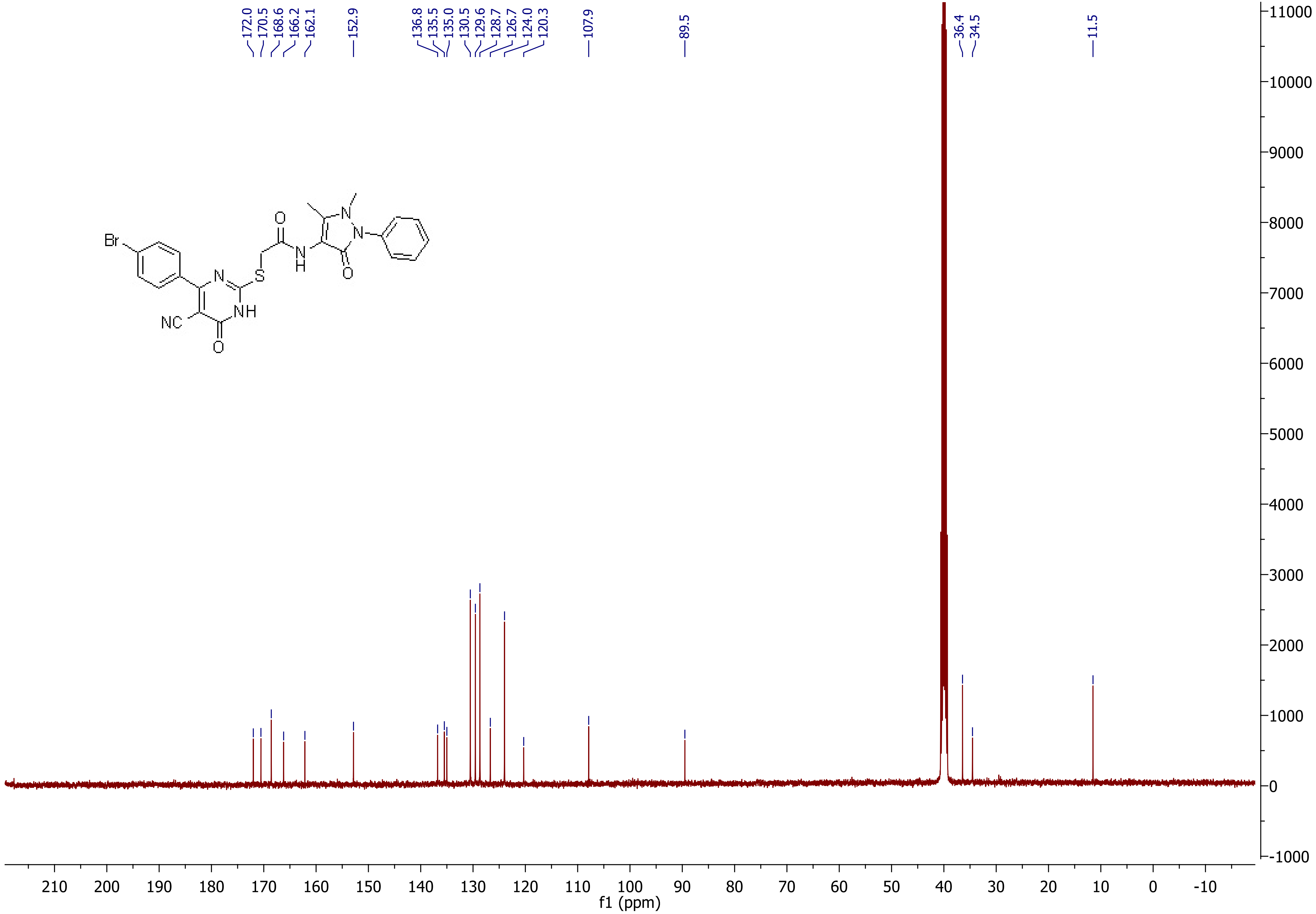
172.0
170.5
168.6
166.2
162.1
152.9
136.8
135.5
135.0
130.5
129.6
128.7
126.7
124.0
120.3
107.9
89.5
36.4
34.5
11.5
Br
N
S
NH
NC
O
O
N
H
N
N
O
11000
10000
9000
8000
7000
6000
5000
4000
3000
2000
1000
0
-1000
210 200 190 180 170 160 150 140 130 120 110 100 90 80 70 60 50 40 30 20 10 0 -10
f1 (ppm)

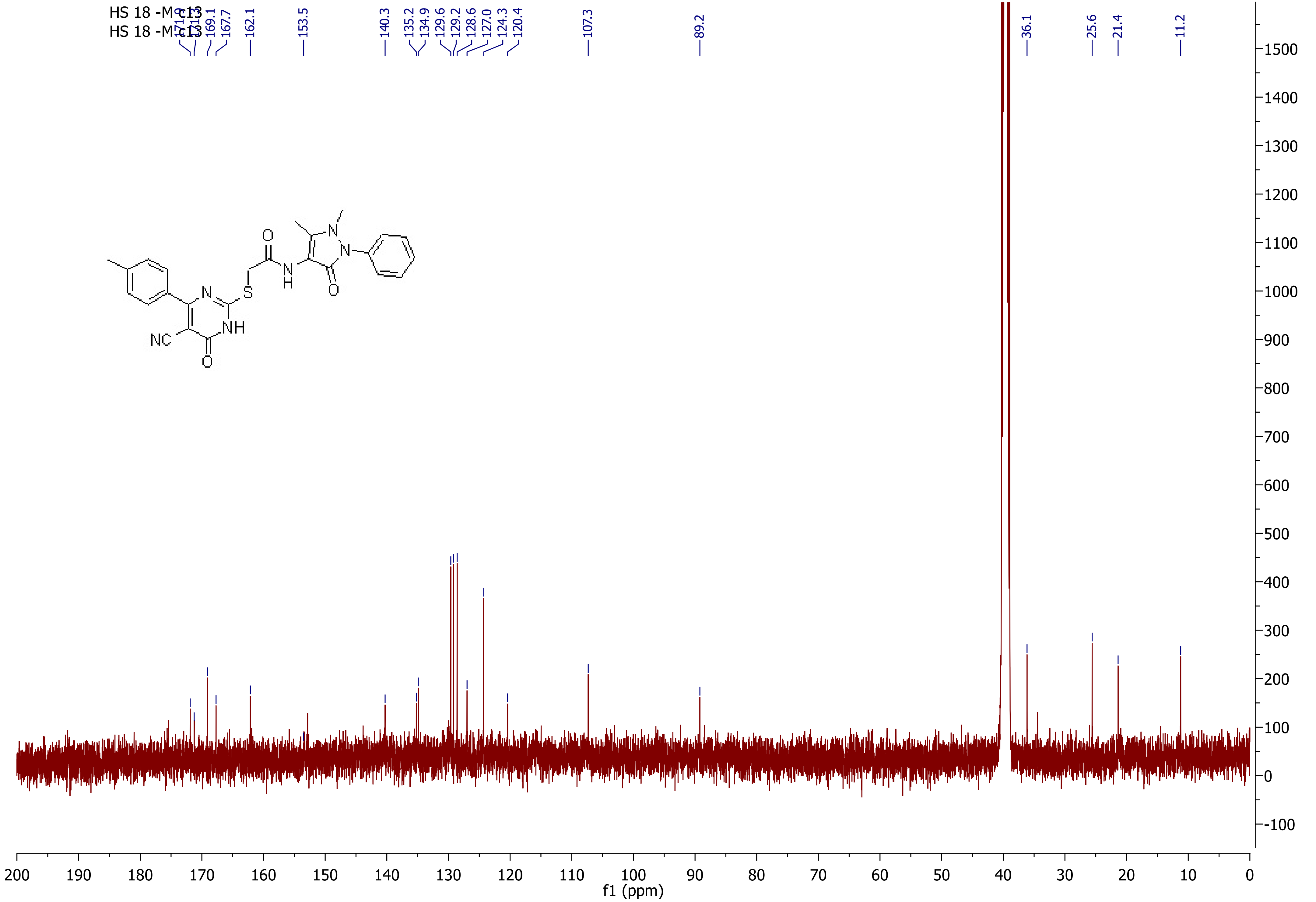
HS 18 -Mc13
169.1
167.7
162.1
153.5
140.3
135.2
134.9
129.6
129.2
128.6
127.0
124.3
120.4
107.3
89.2
36.1
25.6
21.4
11.2
NC
N
NH
S
O
N
H
f1 (ppm)

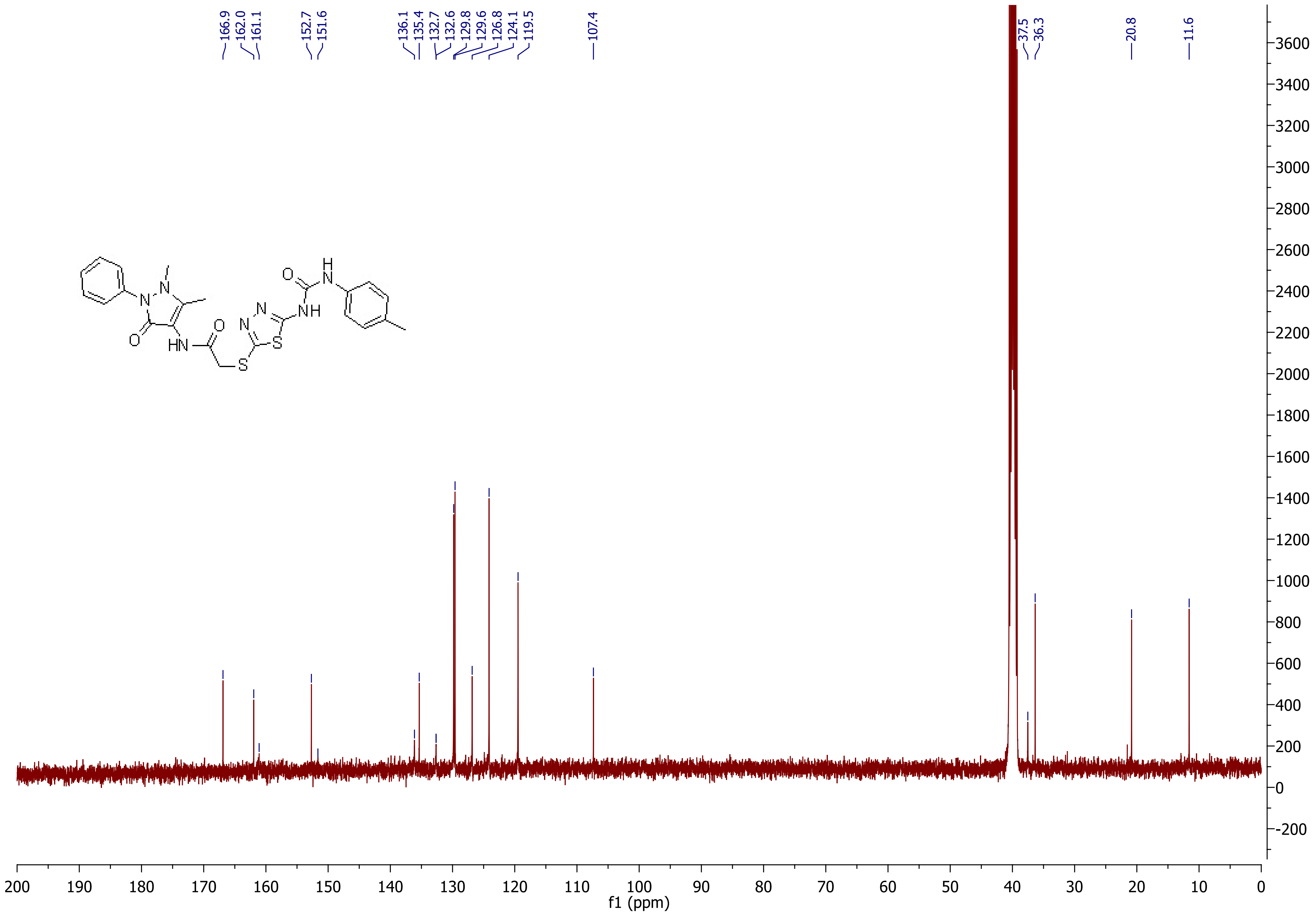

166.9
162.0
161.1
152.7
151.6
136.1
135.4
132.7
132.6
129.8
129.6
126.8
124.1
119.5
107.4
37.5
36.3
20.8
11.6
f1 (ppm)

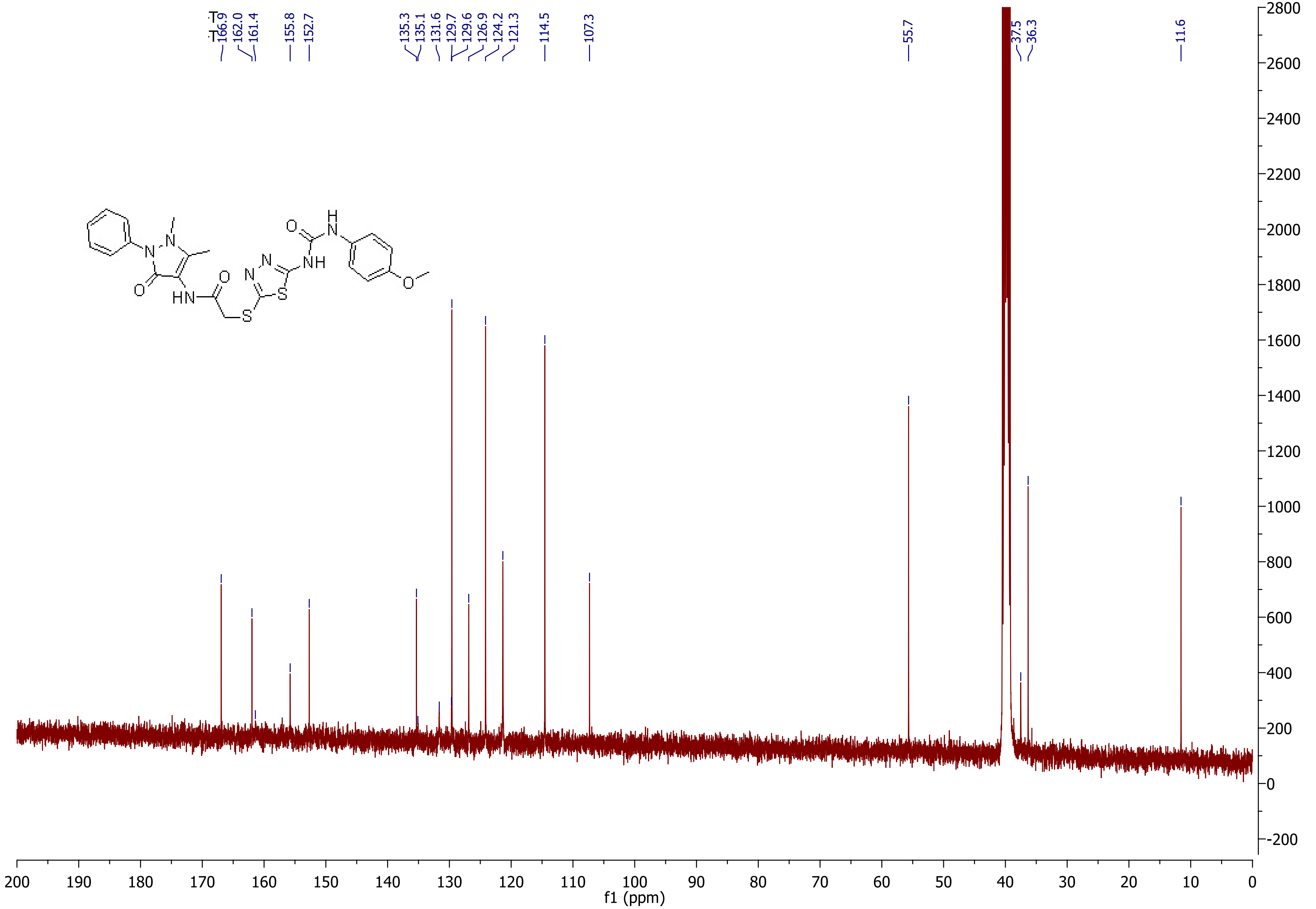
166.9
162.0
161.4
155.8
152.7
135.3
135.1
131.6
129.7
129.6
126.9
124.2
121.3
114.5
107.3
55.7
37.5
36.3
11.6
f1 (ppm)

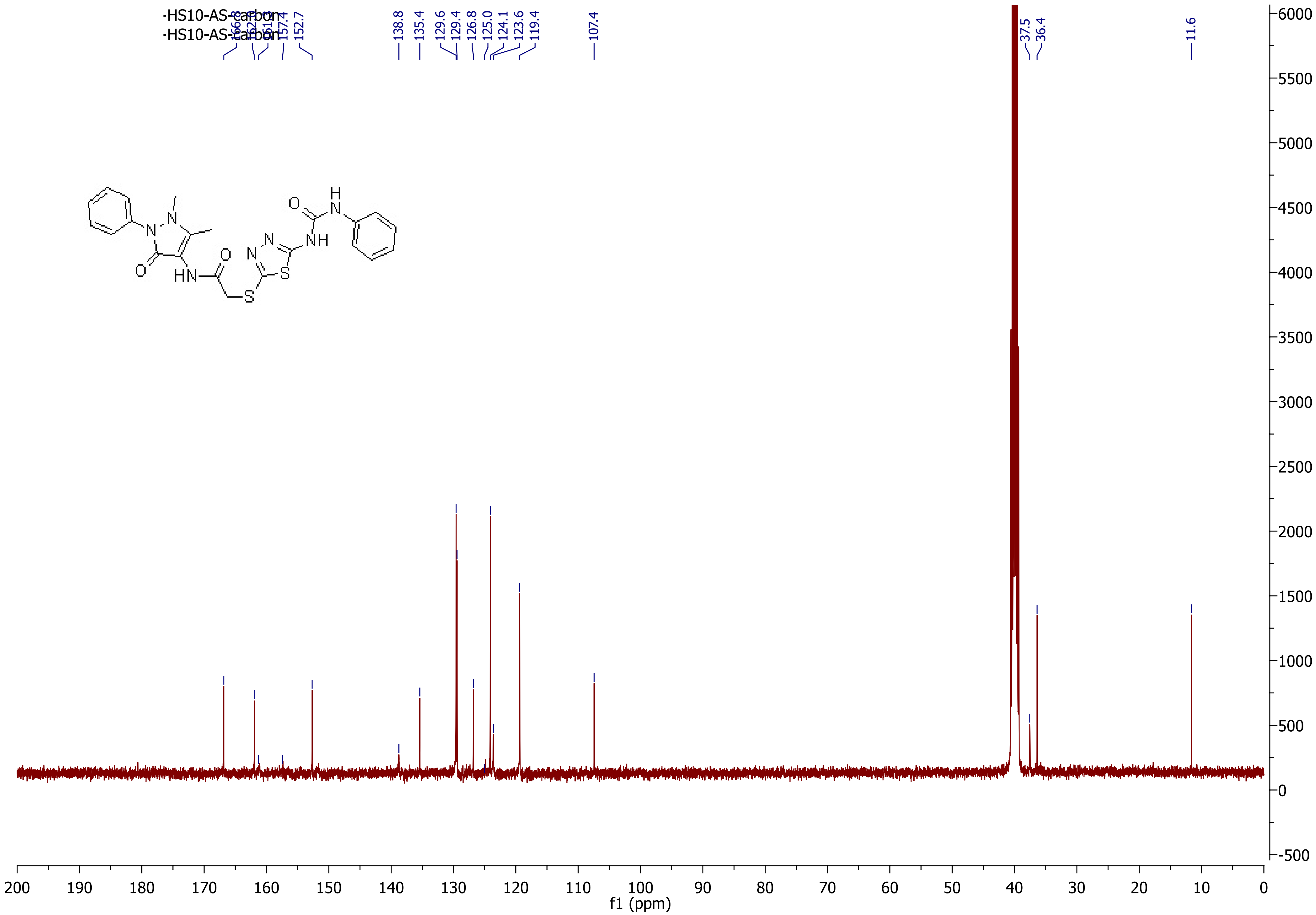

-HS10-AS-carbon
-HS10-AS-carbon
166.8
162.0
161.3
157.4
152.7
138.8
135.4
129.6
129.4
126.8
125.0
124.1
123.6
119.4
107.4
37.5
36.4
11.6
O
N
N
HN
O
N
N
S
S
NH
O
H
N
6000
5500
5000
4500
4000
3500
3000
2500
2000
1500
1000
500
0
-500
200
190
180
170
160
150
140
130
120
110
100
90
80
70
60
50
40
30
20
10
0
f1 (ppm)

CH3-oxa

IC_{50} = 86.92
R^2 = 0.8791

Best-fit values	
LogIC50	1.939
HillSlope	-0.3184
IC50	86.92

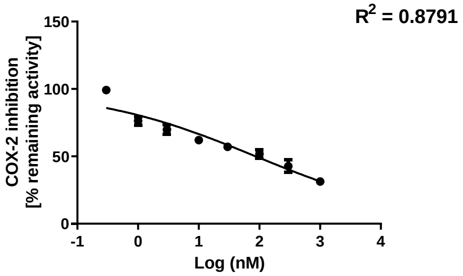

Cl

IC_{50} = 53.76
R^2 = 0.9263

Best-fit values	
LogIC50	1.730
HillSlope	-0.2859
IC50	53.76

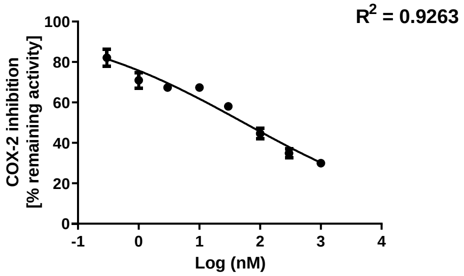

Br-oxa

IC_{50} = 69.79
R^2 = 0.8946

Best-fit values	
LogIC50	1.844
HillSlope	-0.3652
IC50	69.79

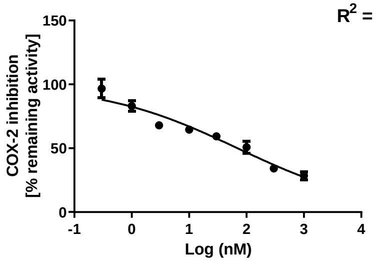

HS40

IC_{50} = 65.54
R^2 = 0.8594

Best-fit values	
LogIC50	1.817
HillSlope	-0.3340
IC50	65.54

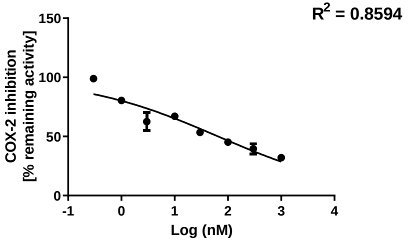

HS23

IC_{50} = 103.7
R^2 = 0.8585

Best-fit values	
LogIC50	2.016
HillSlope	-0.2866
IC50	103.7

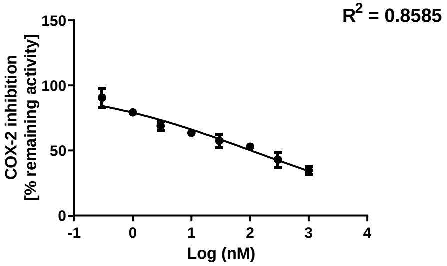

HS10

IC_{50} = 154.8
R^2 = 0.9286

Best-fit values	
LogIC50	2.190
HillSlope	-0.3826
IC50	154.8

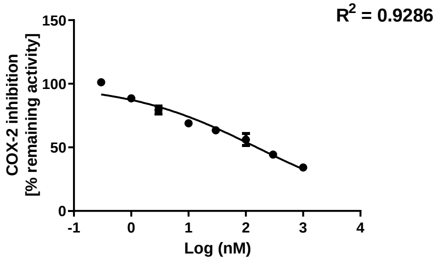

HS27

IC_{50} = 86.10
R^2 = 0.8489

Best-fit values	
LogIC50	1.935
HillSlope	-0.3558
IC50	86.10

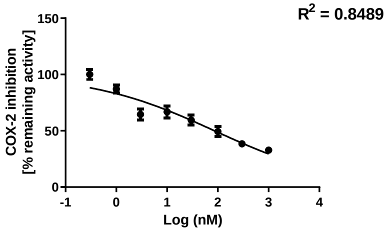

HS13

IC_{50} = 169.0
R^2 = 0.8359

Best-fit values	
LogIC50	2.228
HillSlope	-0.3273
IC50	169.0

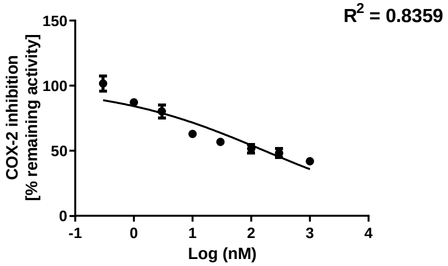

HS18

IC_{50} = 88.62
R^2 = 0.8752

Best-fit values	
LogIC50	1.948
HillSlope	-0.3794
IC50	88.62

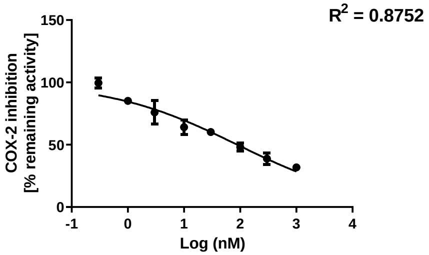

HS16

IC_{50} = 119.8
R^2 = 0.9289

Best-fit values	
LogIC50	2.079
HillSlope	-0.3650
IC50	119.8

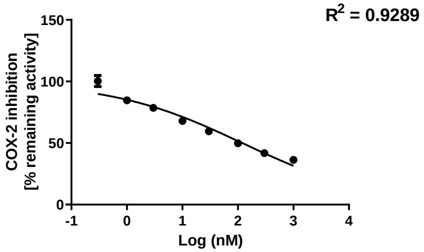

HS17

IC_{50} = 129.8
R^2 = 0.9145

Best-fit values	
LogIC50	2.113
HillSlope	-0.3953
IC50	129.8

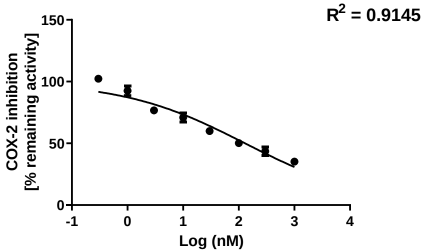

HS11

IC_{50} = 68.74
R^2 = 0.8776

Best-fit values	
LogIC50	1.837
HillSlope	-0.3543
IC50	68.74

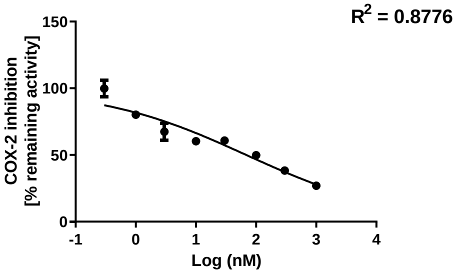

Best-fit values	
LogIC50	1.662
HillSlope	-0.3735
IC50	45.93

Celecoxib

IC_{50} = 45.93

R^2 = 0.8742

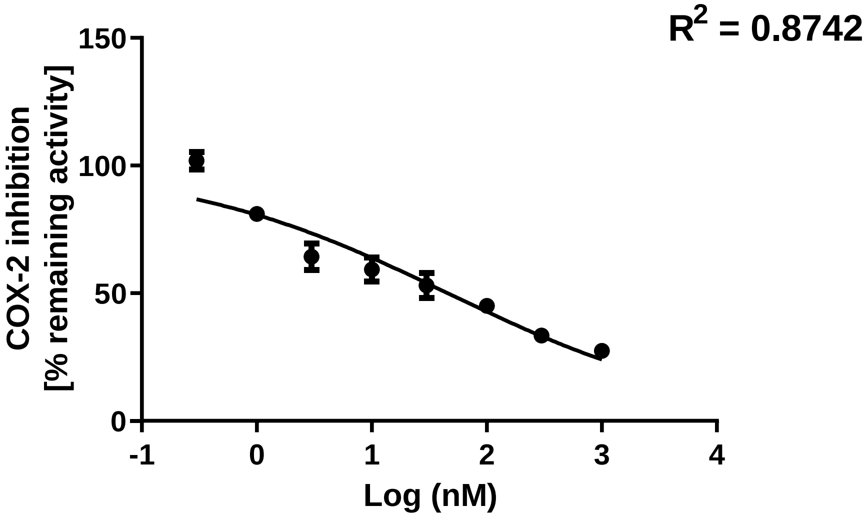

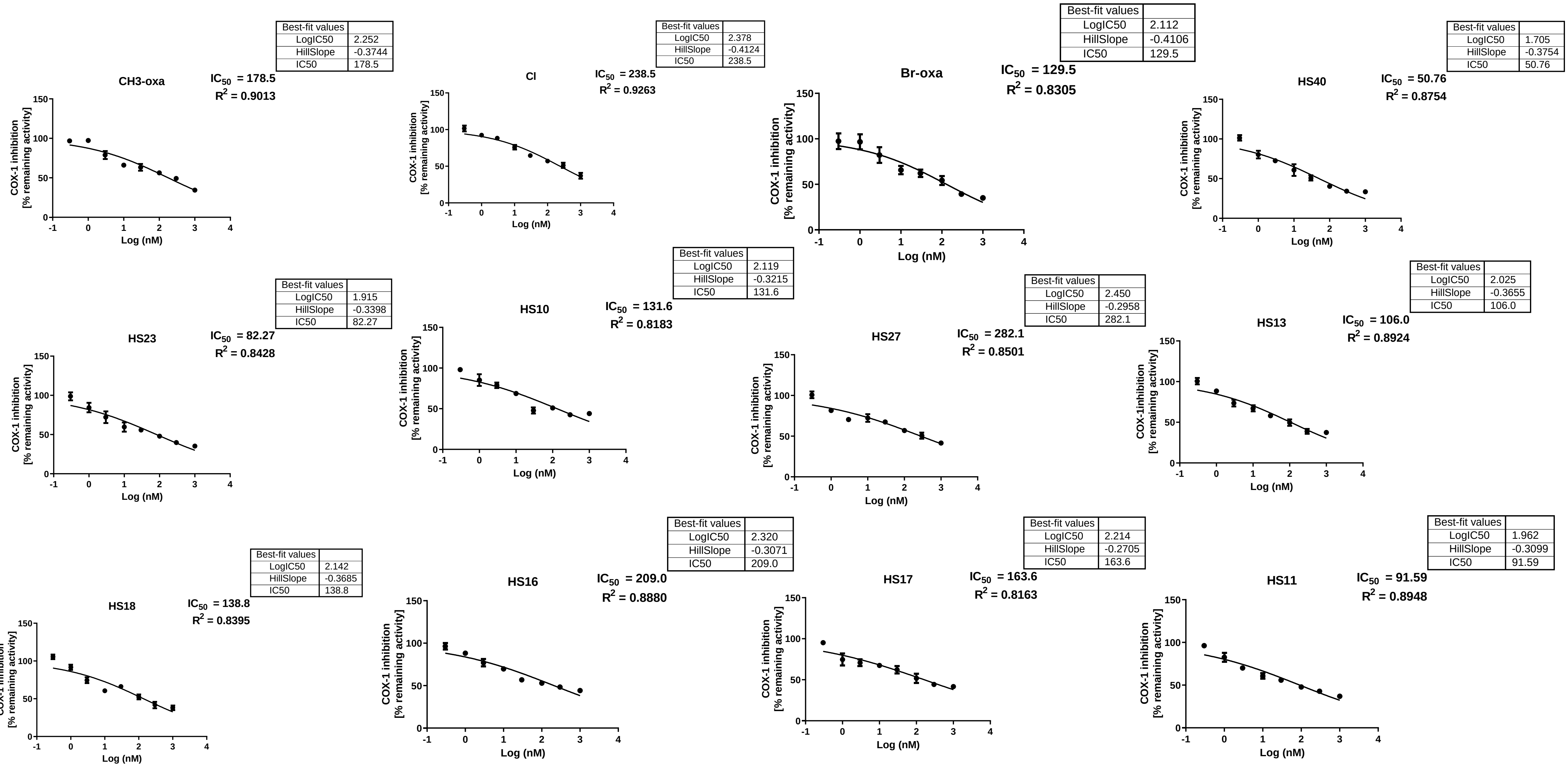
CH3-oxa
IC50 = 178.5
R2 = 0.9013
Best-fit values
LogIC50 2.252
HillSlope -0.3744
IC50 178.5
Cl
IC50 = 238.5
R2 = 0.9263
Best-fit values
LogIC50 2.378
HillSlope -0.4124
IC50 238.5
Br-oxa
IC50 = 129.5
R2 = 0.8305
Best-fit values
LogIC50 2.112
HillSlope -0.4106
IC50 129.5
HS40
IC50 = 50.76
R2 = 0.8754
Best-fit values
LogIC50 1.705
HillSlope -0.3754
IC50 50.76
HS23
IC50 = 82.27
R2 = 0.8428
Best-fit values
LogIC50 1.915
HillSlope -0.3398
IC50 82.27
HS10
IC50 = 131.6
R2 = 0.8183
Best-fit values
LogIC50 2.119
HillSlope -0.3215
IC50 131.6
HS27
IC50 = 282.1
R2 = 0.8501
Best-fit values
LogIC50 2.450
HillSlope -0.2958
IC50 282.1
HS13
IC50 = 106.0
R2 = 0.8924
Best-fit values
LogIC50 2.025
HillSlope -0.3655
IC50 106.0
HS18
IC50 = 138.8
R2 = 0.8395
Best-fit values
LogIC50 2.142
HillSlope -0.3685
IC50 138.8
HS16
IC50 = 209.0
R2 = 0.8880
Best-fit values
LogIC50 2.320
HillSlope -0.3071
IC50 209.0
HS17
IC50 = 163.6
R2 = 0.8163
Best-fit values
LogIC50 2.214
HillSlope -0.2705
IC50 163.6
HS11
IC50 = 91.59
R2 = 0.8948
Best-fit values
LogIC50 1.962
HillSlope -0.3099
IC50 91.59
COX-1 inhibition [% remaining activity]
Log (nM)

Best-fit values	
LogIC50	2.491
HillSlope	-0.3626
IC50	309.6

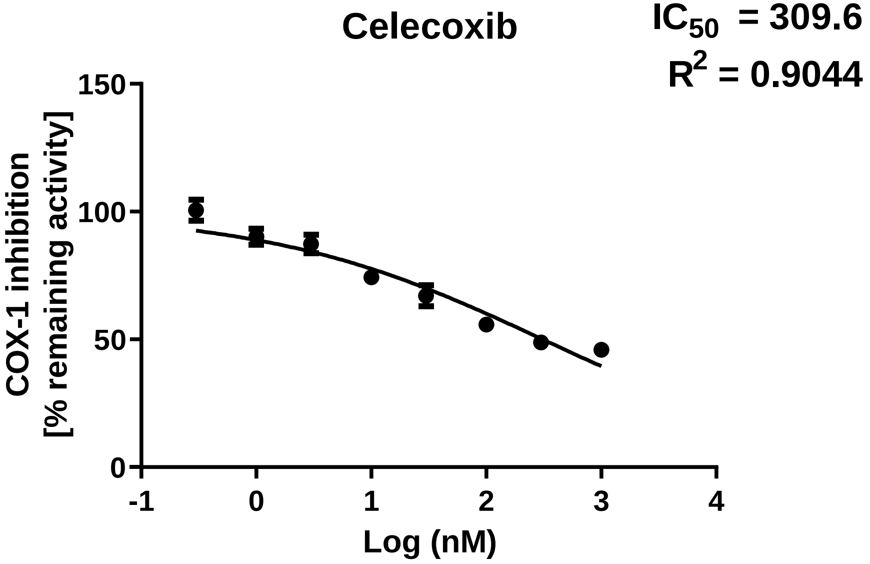

Validation of the docking protocol

In order to validate the docking protocol, we conducted computational experiments based on redocking procedures. In particular, considering that the crystal structure of *h*COX-1 did not present any ligand in the ligand binding site, to assess if the structure was suitable for docking studies, we compared the docking output of the human enzyme with that found for the ovine enzyme using a reference ligand (celecoxib). First of all we assesed the performance of the docking protocol by redocking celecoxib into the ovine enzyme. The output is reported in Figure S1.

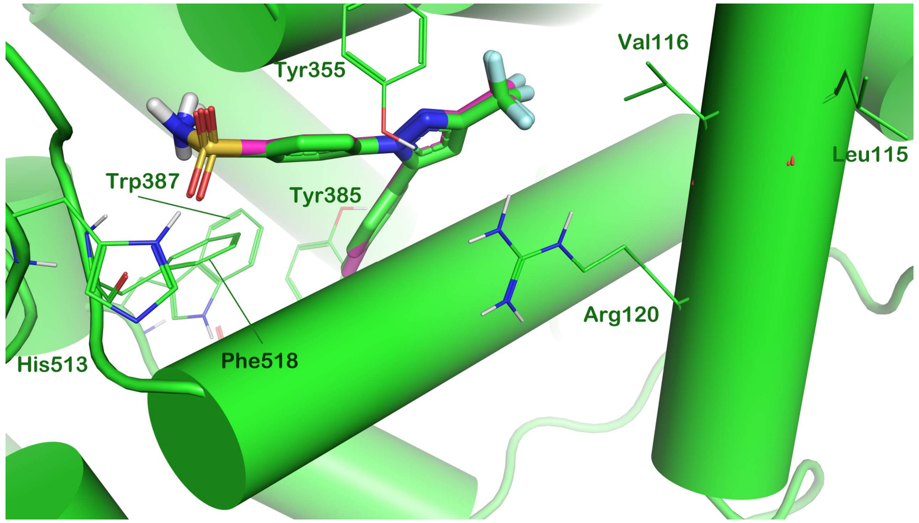

Figure S1. Superposition between the crystal structure of ovine COX-1 (green cartoon) in complex with celecoxib (magenta sticks) (PDB ID 3KK6) and the docked pose of celecoxib (green sticks) as found by applying the docking protocol (RMSD 0.481 Å).

As highlighted by the pictorial representation, the docking protocol is able to correctly accommodate the reference compound into the binding site with a small RMSD value, with a docking score of -8.328 kcal/mol. Accordingly, since we would like to use the human enzyme, but a unique structure in the PDB database is the enzyme without any ligand in the binding site, we evaluated if the docking protocol could able to retrieve the described binding mode in ovine enzyme also considering the human enzyme. By applying the same docking protocol and using the human enzyme (PDB ID 6Y3C), we obtained a very similar output that is reported in Figure S2.

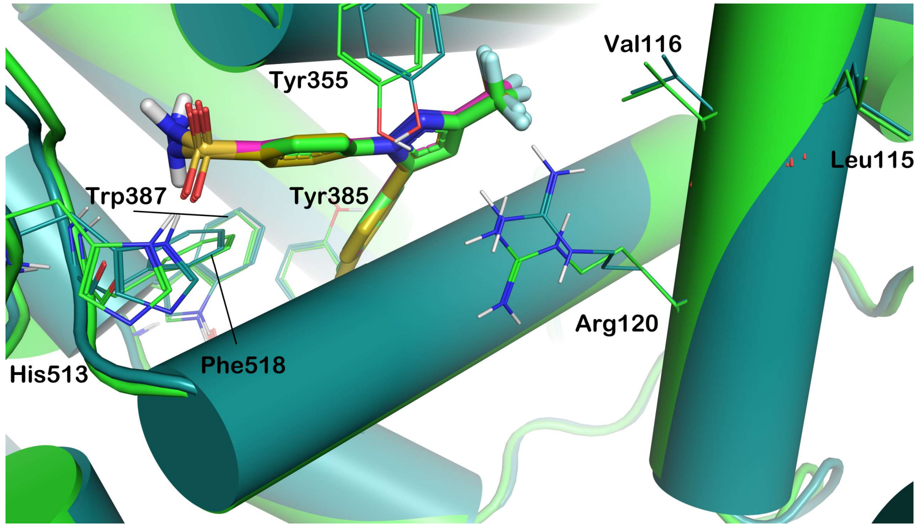

Figure S2. Superposition between the crystal structure of ovine COX-1 (green cartoon) in complex with celecoxib (magenta sticks) (PDB ID 3KK6), the docked pose of celecoxib (green sticks) into the ovine enzyme, and the docked pose of celecoxib (gold sticks) into the human enzyme (deepteal cartoon) (PDB ID 6Y3C) as found by applying the docking protocol.

As illustrated in Figure S2, the docking protocol was able to correctly accommodate the celecoxib also into the human enzyme without any difference in the binding mode. Furthermore, also the docking score found when the reference compound, celecoxib, was docked into the human enzyme is very close to that found for the ovine enzyme, with a value of -8.211 kcal/mol.

Accordingly, the docking protocol was able to correctly accommodate the reference compound into *h*COX-1.

Regarding the validation of the docking protocol for the hCOX-2, we used the crystal structure of the enzyme in complex with rofecoxib (PDB ID 5KIR). The output of the validation procedure is reported in Figure S3.

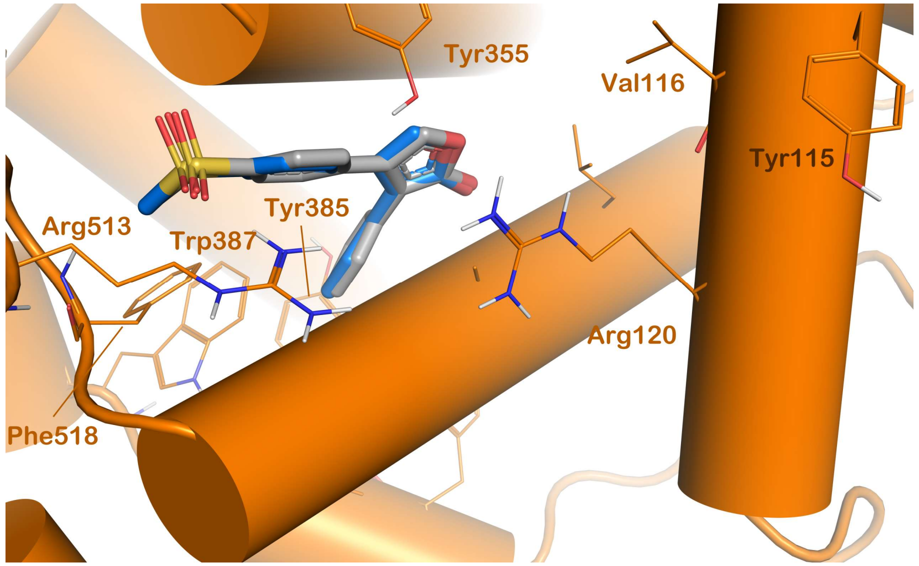

Figure S3. Superposition between the crystal structure of *h*COX-2 (orange cartoon) in complex with rofecoxib (magenta sticks) (PDB ID 5KIR) and the docked pose of celecoxib (green sticks) as found by applying the docking protocol (RMSD 0.169 Å).

As highlighted in the Figure S3, the docking protocol was able to correclty accommodate the rofecoxib into the binding site according to the crystallized binding mode with a very small RMSD and with a docking score of -9.424 kcal/mol. Furthermore, to evaluate the docking score of celecoxib into *h*COX-2, we conducted a docking study applying the same protocol. We found that the docking score of celecoxib into the human enzyme was of -9.631 kcal/mol.

In summary, the docking protocol was able to correctly accommodate the reference drugs into the human enzymes (*h*COX-1 and *h*COX-2) with small differences with respect to the crystal structures.
